# Efficiently targeted therapy of glioblastoma xenograft via multifunctional biomimetic nanodrugs

**DOI:** 10.1186/s40824-022-00309-y

**Published:** 2022-12-02

**Authors:** Zhipeng Yao, Xiaochun Jiang, Hong Yao, Yafeng Wu, Fan Zhang, Cheng Wang, Chenxue Qi, Chenhui Zhao, Zeyu Wu, Min Qi, Jia Zhang, Xiaoxiang Cao, Zhichun Wang, Fei Wu, Chengyun Yao, Songqin Liu, Shizhang Ling, Hongping Xia

**Affiliations:** 1 School of Chemistry and Chemical Engineering & Interdisciplinary Innovation Institute for Medicine and Engineering, Southeast University, Nanjing, 211189 China; 2 The Translational Research Institute for Neurological Disorders, Department of Neurosurgery of Wannan Medical College, the First Affiliated Hospital of Wannan Medical College (Yijishan Hospital of Wannan Medical College), Wuhu, 241001 People’s Republic of China; 3 Department of Pathology, Nanjing Drum Tower Hospital & Drum Tower Clinical College & Key Laboratory of Antibody Technique of National Health Commission & Jiangsu Antibody Drug Engineering Research Center, Nanjing Medical University, Nanjing, 211166 China; 4 The Department of Cancer Biotherapy Center& The Institute of Cancer Research, The Third Affiliated Hospital of Kunming Medical University & The Cancer Hospital of Yunnan province, Kunming, 650000 China; 5 Department of Gynecologic Oncology, Cancer Hospital of Shantou University Medical College, Shantou, 515041 China; 6 Jiangsu Cancer Hospital, The Affiliated Cancer Hospital of Nanjing Medical University, Jiangsu Institute of Cancer Research, Nanjing, 210009 China

**Keywords:** Glioblastoma, Doxorubicin, Biomimetic, Nanodrug, Antitumor

## Abstract

**Background:**

Glioblastoma multiforme (GBM) is a fatal malignant primary brain tumor in adults. The therapeutic efficacy of chemotherapeutic drugs is limited due to the blood-brain barrier (BBB), poor drug targeting, and short biological half-lives. Multifunctional biomimetic nanodrugs have great potential to overcome these limitations of chemotherapeutic drugs.

**Methods:**

We synthesized and characterized a biomimetic nanodrug CMS/PEG-DOX-M. The CMS/PEG-DOX-M effectively and rapidly released DOX in U87 MG cells. Cell proliferation and apoptosis assays were examined by the MTT and TUNEL assays. The penetration of nanodrugs through the BBB and anti-tumor efficacy were investigated in the orthotopic glioblastoma xenograft models.

**Results:**

We showed that CMS/PEG-DOX-M inhibited cell proliferation of U87 MG cells and effectively induced cell apoptosis of U87 MG cells. Intracranial antitumor experiments showed that free DOX hardly penetrated the BBB, but CMS/PEG-DOX-M effectively reached the orthotopic intracranial tumor through the BBB and significantly inhibited tumor growth. Immunofluorescence staining of orthotopic tumor tissue sections confirmed that nanodrugs promoted apoptosis of tumor cells. This study developed a multimodal nanodrug treatment system with the enhanced abilities of tumor-targeting, BBB penetration, and cancer-specific accumulation of chemotherapeutic drugs by combining chemotherapy and photothermal therapy. It can be used as a flexible and effective GBM treatment system and it may also be used for the treatment of other central nervous systems (CNS) tumors and extracranial tumors.

**Supplementary Information:**

The online version contains supplementary material available at 10.1186/s40824-022-00309-y.

## Background

Glioblastoma multiforme (GBM) is a common malignancy in the central nervous system (CNS) for which the current standard of care is surgery followed by chemotherapy and radiation therapy [1, 2]. However, the median survival of GBM patients receiving the current standard of care was 14.6 months, and the 5-year survival rate was only 9.8 % [3]. A large part of this dismal prognosis is due to the existence of the blood-brain barrier (BBB) that prevents most chemotherapeutic drugs from entering the brain [4, 5]. In addition, the choice of chemotherapeutic drugs for the treatment of GBM is limited, and the chemotherapeutic drugs available produce obvious side effects and cause severe systemic toxicity to normal tissues and organs [6, 7]. In contrast, nanodrugs have a lot of potentials to overcome these limitations. The advantages of nanodrugs include prolonged drug circulation time, improved specific drug distribution, and reduced drug side effects [8]. Nanodrugs have been becoming one of the most promising areas of drug development for tumor and other major diseases [9, 10]. However, nanodrugs in the formulation of nanoparticles are exogenous substances, and their recognition by the immune system and their interception by the liver and kidneys seriously limit their clinical applications [11]. Therefore, there is a need to design and develop more biocompatible nanodrugs, among which biomimetic nanodrugs coated with active cell membranes are receiving increasing attention [12].

Cell membrane wrapping strategies have been applied to nanoparticles made of all kinds of materials (nanomaterials). Cell membranes of different cell types have been used to coat nanoparticles. For example, a leukocyte membrane was used to coat silica nanoparticles to penetrate the endothelium [13], a platelet membrane was utilized to coat nanomaterials to target tumor cells [14], and erythrocyte membrane coating increased the circulation time of nanoparticles [15]. Among the biomaterials found in cell membranes, the membrane proteins of tumor cell membranes have structure and adhesion properties that allow them to combine with homologous cells [16]. Biomimetic nanoparticles are formed by wrapping nanoparticles in the cell membrane of tumor cells. Nanodrugs camouflaged by tumor cell membranes can obtain the same surface properties and functions as homologous cell membranes, which enable tumor cell membrane-encapsulated nanodrugs to possess tumor-targeting properties [17]. Biomimetic nanoparticles were reported by wrapping nanoparticles in the cell membrane of MCF-7 cells, a human breast cancer cell line [16]. The biomimetic nanoparticles exhibited homologous targeting of breast cancer cells. They significantly promoted the endocytosis of nanodrugs into breast cancer cells, thereby explicitly causing targeted tumor accumulation of nanodrugs in vivo [16]. It was reported that rat glioma cell C6 cell membrane-camouflaged nanosuspensions could penetrate the BBB and target tumors through immune escape and homotypic binding of tumor cell membranes [18]. Therefore, tumor cell membrane-encapsulated nanodrugs have tumor-specific homologous targeting, which is particularly suitable for effective targeted tumor therapy.

The tumor microenvironment (TME) of solid tumors is characterized by hypoxia, high H_2_O_2_ content, low pH, and high glutathione (GSH) levels [19]. Therefore, combination therapy and modulation of TME are essential for solid tumor treatment. Recently, nanomaterials based on the structure of polyvalent metal ions (such as Mn^2+/4+^, Fe^2+/3+^, Cu^1+/2+^) have been found to possess enzyme-like activities that mimic the functions of a variety of natural enzymes, thereby improving the tumor treatment efficacy of chemotherapeutics by modulating TME [20–22]. Among them, copper-molybdenum sulfide (Cu_2_MoS_4_) (CMS) has attracted great attention for its excellent enzyme-like function [23]. CMS contains polyvalent metal ions (Cu^1+/2+^, Mo^4+/2+^) that have strong absorption of heat in the near-infrared region (NIR), allowing it to have potential antitumor effects [24]. Doxorubicin (DOX), an anthracycline topoisomerase inhibitor, is a common antitumor chemotherapeutic drug considered one of the most effective chemotherapy drugs, and the US Food and Drug Administration (FDA) has approved it to be used for a variety of tumors [25]. The antitumor mechanism of DOX is to intercalate within DNA base pairs to break DNA strands and inhibit the synthesis of both DNA and RNA, inducing cell apoptosis and inhibiting tumors [26]. However, due to the insufficient penetration of the BBB, the therapeutic efficacy of DOX in patients with GBM is severely limited. Therefore, developing a DOX delivery system with improved BBB penetration capability, tumor targeting, and tumor-specific drug release is necessary for the effective treatment of GBM.

To generate a multimodal treatment system by taking advantage of the properties of nanoparticles, DOX, and TME modulators, mesoporous CMS nanoparticles of a smaller size and a larger cavity were developed, then modified with PEG and loaded with DOX to make CMS/PEG-DOX nanoparticles. Finally, a U87 MG tumor cell membrane was used to wrap around the surface of CMS/PEG-DOX to prepare a biomimetic nanodrug, CMS/PEG-DOX-M. In this study, CMS containing multivalent metal elements showed various enzyme-like activities that regulate the TME. Both the nanodrug CMS/PEG-DOX and the biomimetic nanodrug CMS/PEG-DOX-M stably released DOX in U87 MG cells, promoting apoptosis, but hardly affected the activity of HA cells, a normal astrocyte cell line. Compared with CMS/PEG-DOX, the biomimetic nanodrug CMS/PEG-DOX-M was more active in targeting tumors, enriching DOX in tumors, and promoting more tumor cell apoptosis, especially after laser irradiation at 808 nm. It is the first time that we have demonstrated that PEG-modified, DOX-loaded, and cell membrane-wrapped CMS nanodrug not only effectively penetrate the BBB, but also achieves the best capability of GBM inhibition. The synergistic treatment of GBM is achieved by combining chemotherapy, photothermal therapy, and TME modulation (Fig. 8, by Figdraw, http://www.figdraw.com).

## Methods

The detail of the experimental section was described in the Supporting Information online.

### Synthesis of CMS/PEG-DOX

CMS/PEGs were prepared. First, the PEGs and CMS were mixed in PBS buffer in a mass ratio of 1:1 and stirred at 4 °C for 12 h. After that, the mixture was centrifuged at 10,000 rpm for 5 min and washed three times with PBS to obtain CMS/PEG. For DOX loading, DOX and CMS/PEG (1 mg·mL^− 1^, 1 mL) were mixed in PBS buffer in a mass ratio of 2:1 and agitated at 4 °C for 12 h. After that, the mixture was centrifuged at 10,000 rpm for 5 min and washed three times with PBS to obtain CMS/PEG-DOX.

### Preparation and characterization of CMS/PEG-DOX-M

The membrane of U87 MG cells was extracted by a membrane protein extraction kit (P0033, Beyotime Biotechnology). In brief, the cells were washed twice with ice-cold PBS in a 10-cm dish, then scraped off with membrane protein extraction reagent, and thawed repeatedly in liquid nitrogen three times. The membrane protein extract was centrifuged at 700 g for 10 min, and the supernatant was then centrifuged at 2000 g for 30 min, and the pellet was the cell membrane preparation. The CMS/PEG-DOX was encapsulated into the U87 MG cell membrane and then sonicated for 10 min in an ice bath (300 W, on for 2 s, off for 3 s) to achieve membrane coating. The mixture was centrifuged at 10,000 rpm for 5 min to remove the excess cell membrane to obtain CMS/PEG-DOX-cell membrane (CMS/PEG-DOX-M) preparation. The membrane proteins of CMS/PEG-DOX-M preparation were resolved by sodium dodecyl sulfate-polyacrylamide gel electrophoresis (SDS-PAGE). U87 MG cell membrane and CMS/PEG-DOX-M samples were loaded into the wells of 10% SDS-PAGE gel. The gel was stained with 12.5% Coomassie brilliant blue solution and was decolorized in the Coomassie blue staining destaining solution for 6 h before imaging.

### Determination of the loading efficiency of CMS/PEG-DOX

The UV-Vis absorbance of DOX of different concentrations (5,10,20,40,80, or160 μg ml^− 1^) was determined according to a standard curve. DOX and CMS/PEG were mixed in PBS buffer at different mass ratios (1,1,2:1,3:1,4:1, or 5:1), stirred at 4 °C for 12 h, and the supernatant was collected after centrifugation at 10,000 rpm for 5 min. The UV-Vis absorption of free DOX in the supernatant was measured, and the content of free DOX in the supernatant was calculated by the standard curve. Finally, the loading efficiency of DOX is calculated by the formula (DOX input - DOX content in the supernatant) / DOX input × 100%.

### Cell culture

Human glioblastoma cell line (U87 MG), mouse cerebral microvascular endothelial cell line (bEnd.3), and human normal astrocyte cell line (HA) were obtained from the Chinese Academy of Sciences Cell Bank (Shanghai, China). All cells were conventionally cultured in DMEM medium supplemented with 10% FBS in a humidified incubator containing 5% CO_2_ and 95% air at 37 °C.

### Xenograft tumor animal models

6-week-old BALB/c nude mice weighing about 18–22 g were obtained from GemPharmatech (Nanjing, China). The mice were housed at an SPF-level vivarium with free access to food and water as well as a 12 h lighting and 12 h dark cycle. All animal studies were conducted in accordance with the guidelines of the National Regulations on the Care and Use of Laboratory Animals in China. 3 × 10^5^ U87 MG-LUC cells were suspended in PBS for mouse intracranial injection [27]. Briefly, an about 1 cm incision of the skin along the midline of the mouse head was made while the mouse was put in the sagittal position, then subcutaneous tissues were bluntly dissected to expose the skull and the pro-fontanelle. Drilling of the skull at 1 mm of the anterior fontanel after the stereotactic apparatus was adjusted and the microsyringe was finely fixed to ascertain that the microsyringe was located at the puncture point, the needle was slowly advanced vertically for 4 mm and then withdrawn by 1 mm so that the tumor cells were implanted in the left caudate nucleus. A bolus of 10 μl single-cell suspension loaded in a microsyringe was slowly injected at a rate of 2 μl/min for 5 min, then the needle was maintained in position for 2 min before it was slowly withdrawn. Mice were allowed to recover but not be used for experiments until the fluorescent intensity of intracranial fluorescein reached 10^5^ p/s/cm^2^/sr.

### In vivo verification of the penetration of nanodrugs through the BBB

Tumor-bearing BALB/c nude mice of U87 MG-LUC were randomly divided into three groups (*n =* 3). 100 μL of DOX (5 mg/kg), CMS/PEG-DOX (15 mg/kg, DOX 5 mg/kg), and CMS/PEG-DOX-M (15 mg/kg, DOX 5 mg/kg) were injected into the tail vein, respectively. The intensity of DOX autofluorescence in the nude mouse brain was detected by the small animal imaging system (IVIS Lumina LT, PerkinElmer) at different time points (2 h, 4 h, 8 h, or 24 h) to analyze whether the nanodrugs passed through the BBB and the degree of nanodrugs release.

### In vivo tumor treatment efficacy

The tumor-bearing BALB/c nude mice of U87 MG-LUC were randomly divided into seven groups (three mice per group): vehicle, CMS/PEG, CMS/PEG + 808, CMS/PEG-DOX, CMS/PEG-DOX + 808, CMS/PEG-DOX-M, and CMS/PEG-DOX-M + 808. Four hours after intravenous injection of the drug, an 808 nm laser (1 W·cm^− 2^) was irradiated for 5 min. We repeated the same NIR treatment every other day. The tumor-bearing mice were anesthetized with 2% isoflurane. The body weight of the mice was measured and recorded every other day. The fluorescence intensity of LUC in nude mice was detected every three days by the small animal imaging system (IVIS Lumina LT, PerkinElmer). After the mice were sacrificed by euthanasia on day 15, the brain tissue was removed and processed to make frozen tissue sections.

### TUNEL assay

Frozen mouse brain tissue sections were fixed with 4% paraformaldehyde for 15 min and washed three times in PBS. TUNEL assay was performed according to the instructions of the TUNEL kit (12,156,792,910, Roche). The slides were finally sealed with anti-fluorescence quenching sealing solution containing DAPI (P0131, Beyotime Technology) and observed under an upright fluorescence microscope (Carl Zeiss Axio Scope Al, Germany). The percentage of apoptosis was measured with ImageJ software.

### Immunohistochemistry

Frozen mouse brain tissue sections were fixed with 4% paraformaldehyde for 15 min and washed three times in PBS, then blocked with 5% BSA for 2 h, incubated with CRT antibody (dilution 1:400, 12,238 T, Cell Signaling Technology) at 4 °C overnight, washed in PBS three times, then incubated with secondary antibody TRITC-labeled sheep anti-rabbit IgG (dilution 1:200, KGAA99, Keygen) at 37 °C for 1 h and washed with PBS for three times. Finally, the slides were sealed with anti-fluorescence quenching sealing solution containing DAPI (P0131, Beyotime Technology) and observed under an upright fluorescence microscope (Carl Zeiss Axio Scope Al, Germany).

## Results

### Characterization of CMS and CMS/PEG-DOX-M

**Fig. 1:**
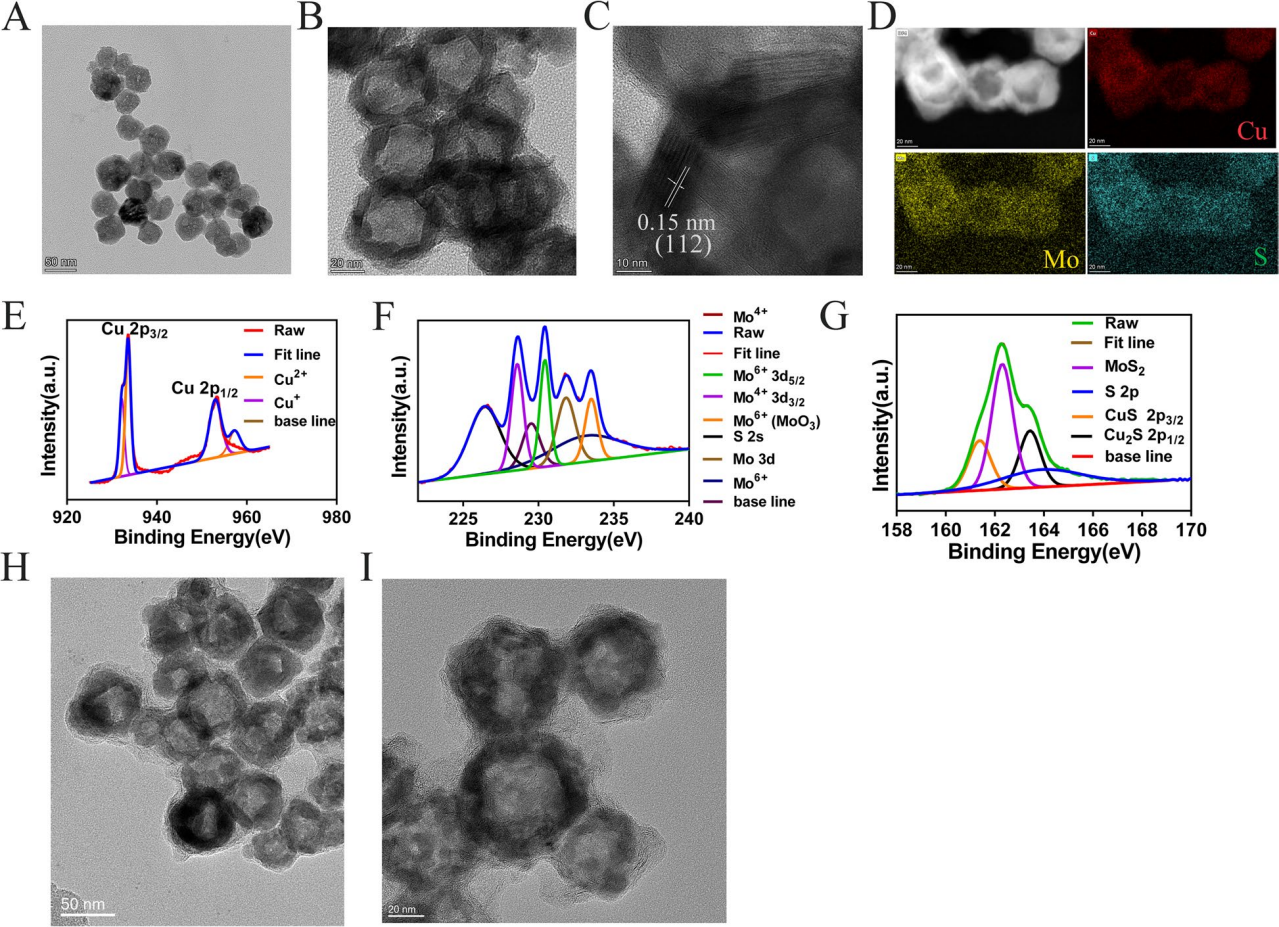
Characterizations of the physicochemical properties of the intermediate product Cu_2_O and the final product CMS. **A** TEM image of Cu_2_O. **B** TEM image of CMS. **C** HR-TEM image of CMS. **D** Elemental mapping of Cu, Mo, and S of CMS. High-resolution XPS spectra of (**E**) Cu 2p, (**F**) Mo 3d, and (**G**) S 2p orbits for the CMS. **H** TEM image of CMS/PEG-DOX-M (scale bar 50 nm). **I** TEM image of CMS/PEG-DOX-M (scale bar 20 nm) .TEM, transmission electron microscopy; CMS, Cu_2_MoS_4_; HR-TEM, high-resolution TEM; XPS, X-ray photoelectron spectroscopy

The physicochemical properties of the intermediate product Cu_2_O and the final product CMS were characterized. The dynamic light scattering (DLS) data show that the average hydrodynamic particle diameters of Cu_2_O and CMS of aqueous solutions are about 90 and 170 nm, respectively (Fig. S1A and S1D, Supporting Information). The images of scanning electron microscopy (SEM) and transmission electron microscopy (TEM) of the synthesized Cu_2_O nanoparticles are shown in Fig. 1A and Fig. S1A-C (Supporting Information). Cu_2_O nanoparticle is in a spherical shape (nanosphere) and its diameter is about 40 nm in size (Fig. 1A, Fig. S1A-C, Supporting Information). CMS nanoparticle was then also characterized by SEM and TEM. These hollow mesoporous nanospheres of CMS are uniform in size, with a diameter of about 50–70 nm (Fig. 1B-C and Fig. S1E-F, Supporting Information). CMS nanospheres were further characterized by high-resolution transmission electron microscopy (HR-TEM). The HR-TEM image of CMS shows that the lattice distance is 0.15 nm, which corresponds to the (112) plane (Fig. 1C). The element mapping from the HR-TEM image clearly shows that the main elements of Cu, Mo, and S are uniformly distributed in the CMS (Fig. 1D). In addition, CMS nanospheres were further characterized by X-ray diffraction (XRD). The XRD pattern of CMS (Fig. S1G, Supporting Information) indicates that CMS is a tetragonal unit cell (space group: I-42 m )[23]. The chemical composition and valence state of CMS were further analyzed by X-ray photoelectron spectroscopy (XPS). The binding energy peaks near 953.01 eV and 932.2 eV belong to Cu 2p1/2 and Cu 2p3/2, respectively, indicating that Cu is composed of Cu^+^ (932.2 eV, 953.01 eV) and Cu^2+^ (933.6 eV, 957.2 eV) composition (Fig. 1E). The peaks at 231.8 eV and 229.5 eV belong to Mo 3d3/2 and Mo 3d5/2, respectively (Fig. 1F). It can be seen that the valence states of Mo are Mo^4+^ (228.5 eV, 229.5 eV) and Mo^6+^ (231.8 eV, 230.4 eV) (Fig. 1F). The peaks with the binding energy of 162.3 eV and 161.4 eV are in line with S 2p1/2 and S 2P3/2, respectively (Fig. 1G), and the corresponding valence state is S^2−^ (Fig. 1G). The existence of Cu and Mo of different valences makes them have Fenton-like, catalase-like, and glutathione peroxidase-like activities.

The morphology and size of the final product CMS/PEG-DOX-M were characterized by TEM, and the CMS/PEG-DOX-M nanodrug are spherical in shape with a diameter of about < 100 nm (Figs. 1 H-I). It can also be clearly seen that there is a membrane structure on the surface of the nanosphere and a certain filling inside the nanosphere (Fig. 1H-I). The results also showed that the nanospheres were successfully loaded with DOX, the U87 MG cell membrane was successfully wrapped in CMS/PEG-DOX, and the CMS/PEG-DOX-M nanodrug was successfully prepared.

### CMS surface modification, DOX loading, and DOX release

The surface of CMS nanospheres was modified by polyethylene glycol 4000 (PEG-4000) through non-covalent conjugation to improve CMS’s dispersion, biocompatibility, and half-life. After PEG-4000 modification, the zeta potential of CMS (CMS/PEG) changed from − 20 mV to − 15 mV (Fig. S2A, Supporting Information).

**Fig. 2:**
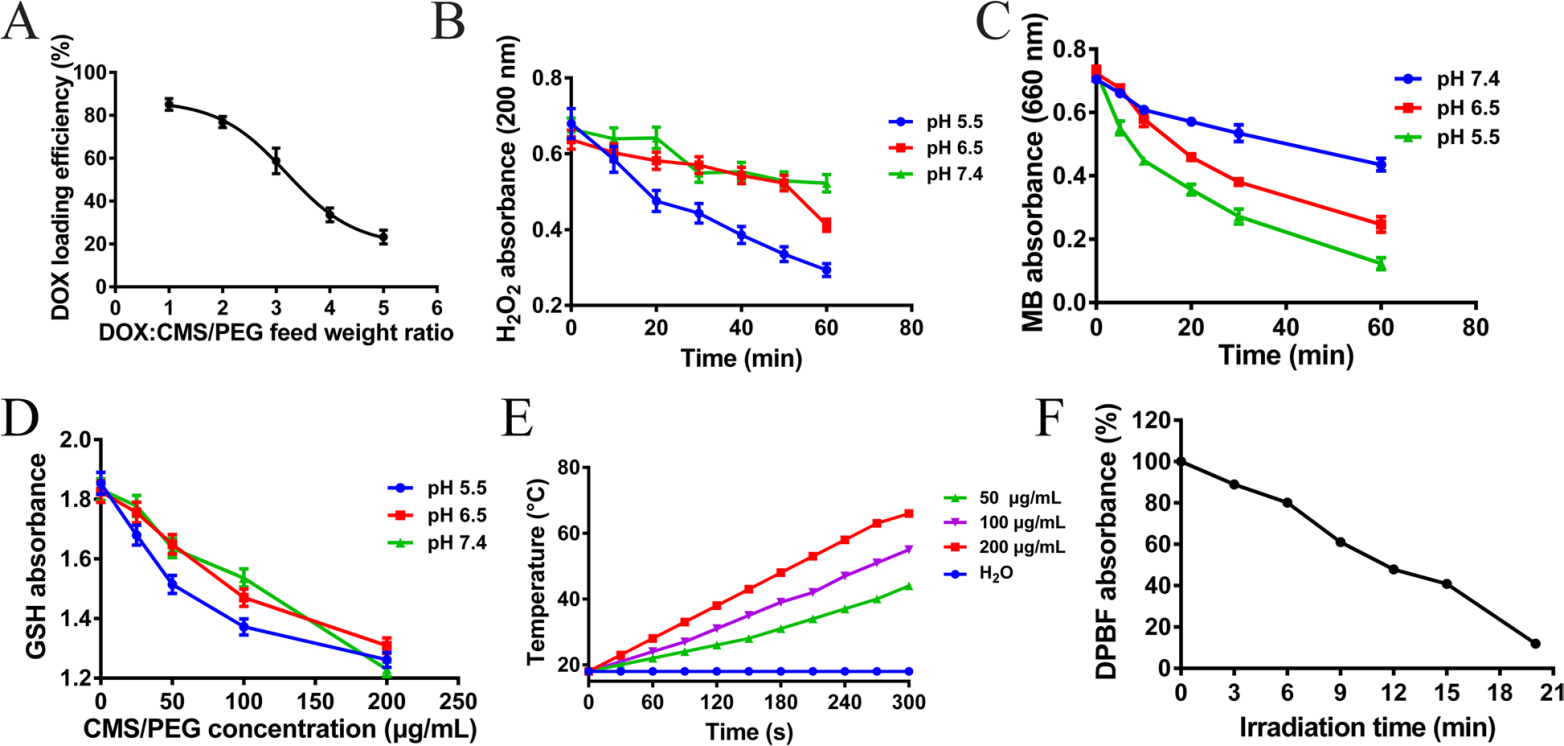
Determinations of the loading efficiency of DOX loaded into CMS/PEG and catalase- and glutathione reductase-like activities of CMS/PEG. **A** DOX-loading efficiency as a function of different DOX:CMS/PEG weight ratios. **B** The maximum UV-Vis absorbance curves of H_2_O_2_ in CMS/PEG solutions of three different pH values over time. **C** The maximum UV-Vis absorbance curves of MB that reacts with hydroxyl free radical (·OH) in the solutions of three different pH values with time. **D** The maximum UV-Vis absorbance curves of GSH as a function of CMS/PEG concentration with three different pH values. **E** The photothermal activity of CMS of different concentrations (200 μL) under infrared laser irradiation (808 nm, 1 W·cm^− 2^) for 5 min. **F** DPBF consumption by ·O_2_^−^ produced from CMS/PEG (50 μg·mL^− 1^) under laser irradiation (808 nm laser, 1 W·cm^− 2^) as a function of irradiation time. DOX, doxorubicin; MB, methylene blue; GSH, glutathione; CMS, Cu_2_MoS_4_; PEG, polyethylene glycol; DPBF, 1,3-diphenylisobenzofuran

CMS/PEG nanoparticles were further characterized by TEM. These nanoparticles of CMS/PEG are uniform in size (data not shown), with a diameter of about 70 nm as nanospheres (Fig. S2B, Supporting Information). TEM imaging also showed that CMS/PEG nanospheres were structurally porous (Fig. 1B; Fig. S2B, Supporting Information). The analysis of the N_2_ adsorption-desorption isotherm and pore size distribution curves of CMS/PEG by the Brunauer-Emmett-Teller (BET) method shows that the average surface area of CMS/PEG is 134.95 m^2^·g^− 1^. The range of pore sizes is between 10 nm and 30 nm, with the predominant pore size at 10 nm (Fig. S2C, Supporting Information), so demonstrating that CMS/PEG nanoparticle has a mesoporous structure. CMS/PEG nanoparticle has abundant pores, which are beneficial to the effective loading of the payload. In addition, no significant aggregation was observed up to 48 h after CMS/PEG nanoparticles were incubated in water, PBS, and cell culture medium (DMEM containing 10% FBS) for the indicated durations (0 h, 12 h, 24 h, or 48 h) (Fig. S3A-B, Supporting Information), showing that CMS/PEG nanospheres have good dispersion and stability, which allow CMS/PEG nanospheres to possess a good potential of drug delivery carrier. When CMS/PEG nanoparticles were loaded with DOX at a weight ratio (DOX: CMS/PEG) of 1:1, the DOX loading rate exceeded 85%, which is relatively high (Fig. 2A and Fig. S2D-E, Supporting Information). The drug-releasing behavior of DOX from CMS/PEG-DOX nanoparticles was then studied in a PBS solution with one of two different pH values. It was found that the rate of DOX release from CMS/PEG-DOX nanospheres was slow at pH 7.4 but rapid at pH 5.5 over time, showing that DOX was released in a pH- and time-dependent manner (Fig. S2F, Supporting Information).

### Detection of catalase-like activity of CMS/PEG

The catalase-like activity of CMS/PEG nanospheres was revealed by the consumption of H_2_O_2_ and the production of visible bubbles of O_2_. The consumption rate of H_2_O_2_ was found by UV-Vis spectrometry and was accelerated in a time-dependent manner and in an acidic pH-dependent manner (Fig. 2B, Fig. S3C-E, Supporting Information). In addition, the production rate of O_2_ observed was increased in a time-dependent manner and in an acidic pH-dependent manner (Fig. S3F, Supporting Information).

It is known that CMS/PEG containing metal ions of multiple valences (Cu^1+/2+^, Mo^4+/6+^) can produce hydroxyl radical (·OH) through a Fenton-like reaction and methylene blue (MB) can be used as an indicator to detect the ·OH generated via the oxidation reaction between MB and ·OH. To examine whether CMS/PEG nanoparticles generate ·OH through a Fenton-like reaction with H_2_O_2_, H_2_O_2_ and MB were added to CMS/PEG nanoparticles of PBS solution with an indicated pH value to react up to 60 min, then MB in the supernatant of CMS/PEG solution was measured by UV-Vis spectrometry at 660 nm. The UV-Vis spectrum shows that the absorbance of MB is decreased in a time-dependent manner and in an acidic pH-dependent manner (Fig. 2C, Fig. S4A-D, Supporting Information), reflecting the amount of ·OH increasingly generated in the same manner. Therefore, these results suggest that CMS can consume H_2_O_2_ in the TME, and simultaneously generate O_2_ and ·OH, promoting cell apoptosis and inhibiting tumor growth.

### Detection of glutathione peroxidase-like activity of CMS/PEG

To examine whether CMS/PEG possesses the ability to react with the most abundant reducing agent, GSH, that exists in the TME, GSH was added to CMS/PEG solution of different concentrations, and then the UV-Vis spectrum of GSH in the supernatant was measured. GSH consumption and color change both reflect the peroxidation of glutathione by CMS/PEG (Fig. 2D, Fig. S4H, Supporting Information) and the consumption of GSH was increased in a pH- and concentration-dependent manner (Fig. 2D; Fig. S4E-H, Supporting Information), demonstrating that CMS/PEG has a peroxidase enzyme-like activity. These data predict that the consumption of GSH in the TME by CMS/PEG is beneficial in destroying the cellular antioxidant defense system of tumors and inhibiting tumor development and growth.

### Photothermal properties of CMS nanomaterials

Based on the broad absorption of CMS to infrared light (Fig. S5A, Supporting Information), we further examined its photothermal properties. It was found that CMS exhibited irradiation dose- and time-dependent photothermal properties under 808 nm laser irradiation (1 W cm^− 2^) (Fig. 2E, Fig. S5D, Supporting Information) and showed excellent photothermal stability (Fig. S5C, Supporting Information), which remained unchanged after repeated cycles of heating and cooling irradiation, suggesting that CMS can be used as a photothermal agent. In addition, in light of the fact that superoxide anion (·O_2_^−^) can be generated when CMS is irradiated with 808 nm laser (1 W· cm^− 2^), so we examined the time course of·O_2_^−^ generated under irradiation using 1,3-diphenylisobenzofuran (DPBF) as a probe that can react with ·O_2_^−^. It was found that the UV-Vis absorption value of DPBF was gradually decreased in a time-dependent illumination manner (Fig. 2F, Fig. S5B, Supporting Information). Taken together, the above results indicate that CMS is a multifunctional nanomaterial.

### Characterization of CMS/PEG-DOX-M

To examine whether the cell membrane can coat CMS/PEG-DOX and the profile of cell membrane proteins is retained after coating, the cell membrane proteins of U87 MG cells and CMS/PEG-DOX-M were characterized by SDS-PAGE. The profile of cell membrane proteins in CMS/PEG-DOX-M is the same as that of U87 MG cell membrane proteins (Fig. S6, Supporting Information), indicating that the U87 MG cell membrane successfully encapsulated CMS/PEG-DOX nanoparticles.

### Biocompatibility of nanodrugs

**Fig. 3:**
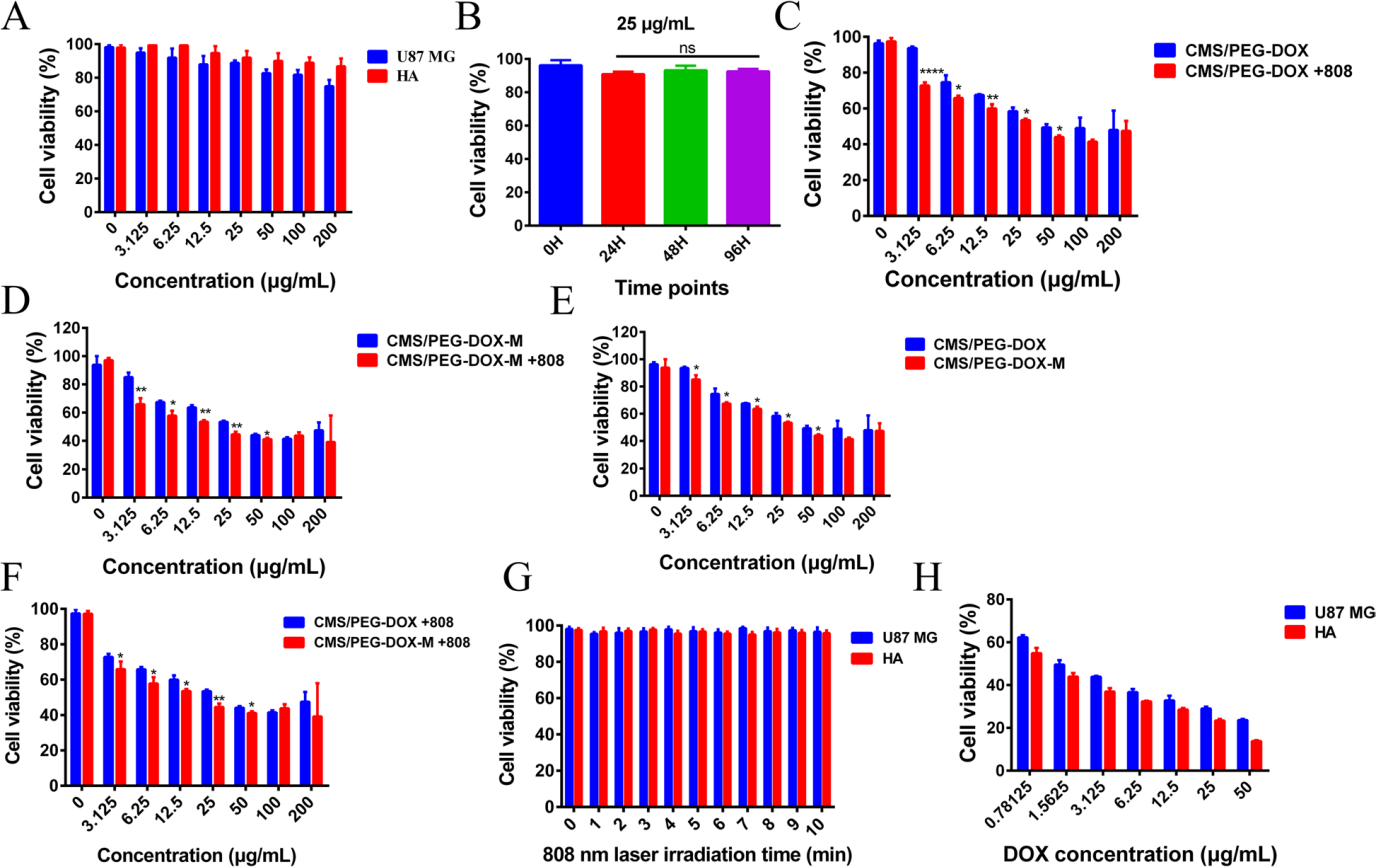
The toxicity of nanodrugs to cells detected by MTT method. **A** Toxicity of CMS/PEG nanoparticles at different concentrations to U87 MG cells and HA cells. **B** Toxicity of CMS/PEG at the concentration of 25 μg/ml to U87 MG cells at the indicated time points. **C** Toxicity of CMS/PEG-DOX at the indicated concentrations to U87 MG cells with or without infrared laser irradiation (808 nm laser, 1 W·cm^− 2^). **D** Toxicity of CMS/PEG-DOX-M at the indicated concentrations to U87 MG cells with or without infrared laser irradiation (808 nm laser,1 W·cm^− 2^). **E** Toxicity of CMS/PEG-DOX or CMS/PEG-DOX-M at the indicated concentrations to U87 MG cells (**F**) Toxicity of CMS/PEG-DOX or CMS/PEG-DOX-M at the indicated concentrations to U87 MG cells with or without infrared laser irradiation (808 nm laser,1 W·cm^− 2^). **G** The toxicity of 808 nm laser (1 W·cm ^− 2^) irradiation for different times to U87 MG or HA cells. **H** Toxicity of free DOX at the indicated concentrations to U87 MG or HA cells. MTT, 3-(4,5-Dimethylthiazol-2-yl)-2,5-diphenyltetrazolium bromide; CMS, Cu_2_MoS_4_; PEG, polyethylene glycol; DOX, doxorubicin; M, cell membrane of U87 MG cells

Without DOX loading, the biocompatibility of CMS/PEG was examined with HA and U87 MG cells. No significant cytotoxicity was observed after CMS/PEG nanoparticles at a concentration from 3.125 to 200 μg/mL were cultured with HA cells for 24 h, and low toxicity was noticed in U87 MG cells at high concentrations from 100 to 200 μg/mL (Fig. 3A). In addition, U87 MG cells treated with 25 μg/mL CMS/PEG were measured for cell viability at different time points (0 h, 24 h, 48 h, or 96 h). No significant cytotoxicity was observed (Fig. 3B). These results show that CMS/PEG nanoparticles as the drug carrier have good biocompatibility. In stark contrast, DOX loading alone enabled CMS/PEG (CMS/PEG-DOX) to kill more than 50% of U87 MG cells and showed even more cytotoxic when U87 MG cells were irradiated with 808 nm laser (1 W·cm^− 2^) (Fig. 3C). Furthermore, the U87 MG cell membrane encapsulation to CMS/PEG-DOX (CMS/PEG-DOX-M), without or with laser irradiation, further enhanced the cytotoxicity of CMS/PEG-DOX to U87 MG cells (Fig. 3D-F). These results suggest that CMS/PEG-DOX itself has a powerful antitumor effect and its cell membrane coating (CMS/PEG-DOX-M) further promotes the antitumor effect on glioma cells. When U87 MG and HA cells were irradiated with only 808 nm laser (1 W·cm^− 2^), almost no cell death occurred even after irradiation for 10 min (Fig. 3G). When U87 MG and HA cells were treated with free DOX, HA cells exhibited higher toxicity than U87 MG cells, even though HA cells died more obviously than U87 MG cells at low concentrations (Fig. 3H).

Considering the dose-effect of CMS/PEG of the above, CMS/PEG at a concentration of 25 μg/mL was selected for the remaining studies. This concentration is well tolerated in a variety of cell lines tested while providing a high enough DOX dosage to observe profound cytotoxicity in tumor cells.

### Cellular uptake of nanodrugs

**Fig. 4:**
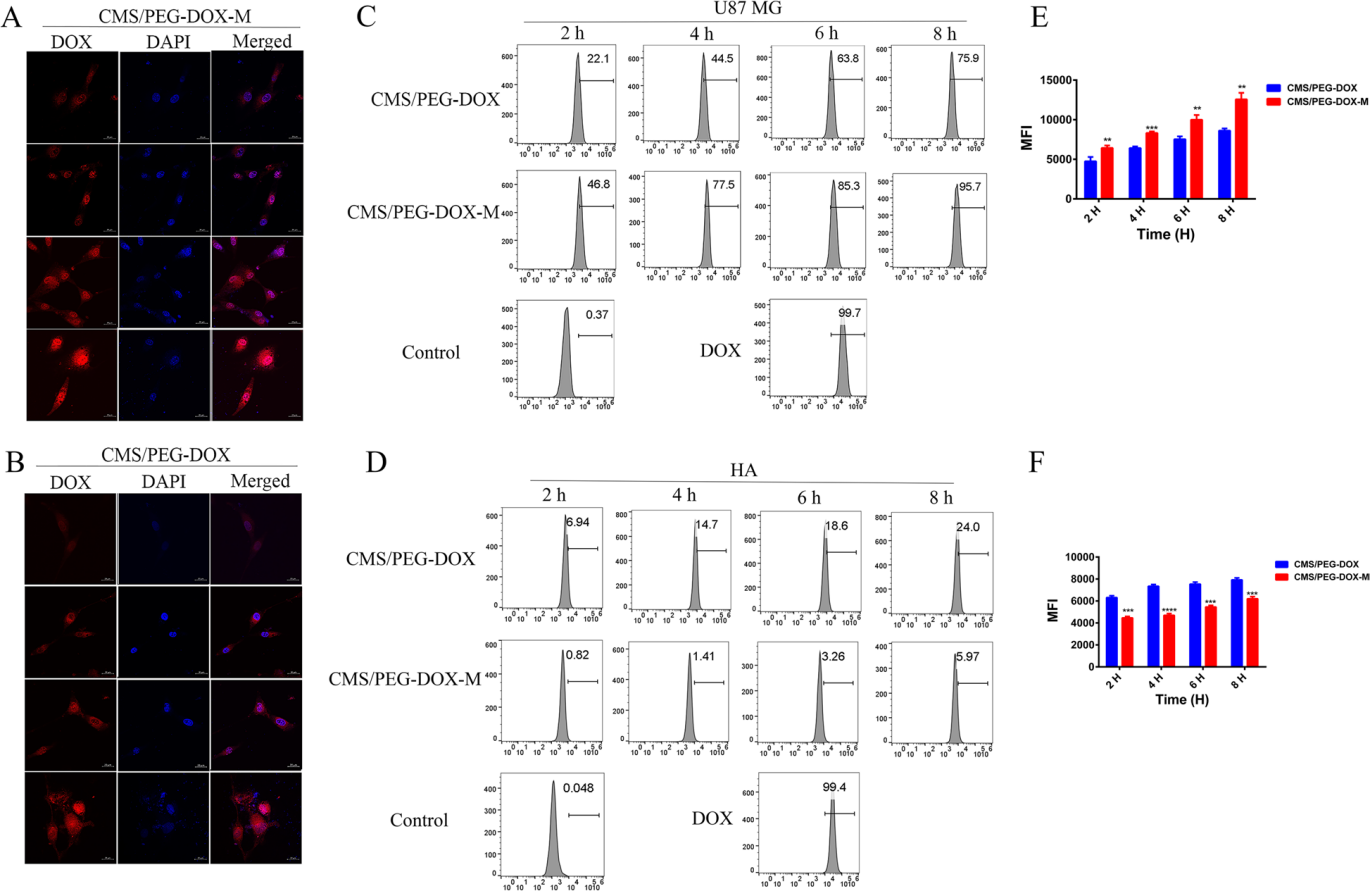
Examinations of the uptake of CMS/PEG-DOX-M or CMS/PEG-DOX into U87 MG and HA cells by confocal microscopy and flow cytometry. **A**, **B** Confocal images of U87 MG cells treated with CMS/PEG-DOX-M (**A**) or CMS/PEG-DOX (**B**) at the indicated time points (scale bar 20 μm). Blue and red colors represent DAPI and DOX fluorescence, respectively. **C**, **D** Flow cytometry images showing the DOX uptake into U87 MG cells (**C**) and HA cells (**D**) treated with either of the indicated treatments at the indicated time points. **E**-**F** MFI of DOX in U87 MG cells (**E**) or HA cells (**F**) treated with CMS/PEG-DOX-M or CMS/PEG-DOX detected by flow cytometry at the indicated time points. CMS, Cu_2_MoS_4_; PEG, polyethylene glycol; DOX, doxorubicin; M, cell membrane of U87 MG cells, DAPI, 4′,6-diamidino-2-phenylindole

To examine the cellular uptake of the nanodrugs that we developed, the U87 MG cells were incubated with CMS/PEG-DOX-M or CMS/PEG-DOX for a different duration of culture time. The confocal images showed that the intracellular fluorescence of DOX was significantly increased with the prolongation of culture time. The amount of uptake of CMS/PEG-DOX-M was much more than that of CMS/PEG-DOX observed in U87 MG cells, consistent with the results of cell viability assays (Fig. 4A-B, Fig. S7A-B, Supporting Information). In addition, DOX fluorescence was found to remain mainly in the cytoplasm after 2 h of culture. Still, DOX accumulated significantly within the nucleus over time, indicating that after the nanoparticles were ingested by the cells, they were decomposed in an acidic environment inside the cells. DOX was gradually released within the cells. The release of nanodrugs in U87 MG cells and HA cells was also detected by flow cytometry. The results were consistent with confocal detection (Fig. 4C-F), and CMS/PEG-DOX-M showed a higher amount of cell uptake than CMS/PEG-DOX (Fig. 4C-E,), which had almost the same uptake profile of free DOX (Fig. 4C). Interestingly, there was little release of DOX from CMS/PEG-DOX or CMS/PEG-DOX-M in particular in the HA cells (Fig. 4D-F), indicating that the intracellular environment of normal cells will not allow the nanodrug to get released even after nanodrug is taken into normal cells. However, almost all of the free DOX entered HA cells and then induced apoptosis of HA cells (Fig. 4D). We also calculated the IC50 of free DOX to U87 MG cytotoxicity. We quantified the amount of DOX released from CMS/PEG-DOX or CMS/PEG-DOX-M nanodrug and ingested by HA and U87 MG cells (Fig. S8A-B, Supporting Information). It was found that the IC50 of free DOX cytotoxicity was 1.034 μg/ml in U87 MG cells (Fig. S8A, Supporting Information). The amount of intracellular DOX released from CMS/PEG-DOX-M in U87 MG was significantly higher than that of CMS/PEG-DOX and IC50 of free DOX in U87 MG cells (Fig. S8B, Supporting Information), and in contrast, the DOX content in HA cells treated with CMS/PEG-DOX or CMS/PEG-DOX-M was very low (Fig. S8B, Supporting Information). The above results demonstrate that the encapsulation of nanodrug with U87 MG cell membrane achieves an effective cell uptake, active cell targeting, and a rapid drug release of nanodrugs in U87 MG cells.

### Nanodrugs affect cell proliferation and apoptosis

**Fig. 5:**
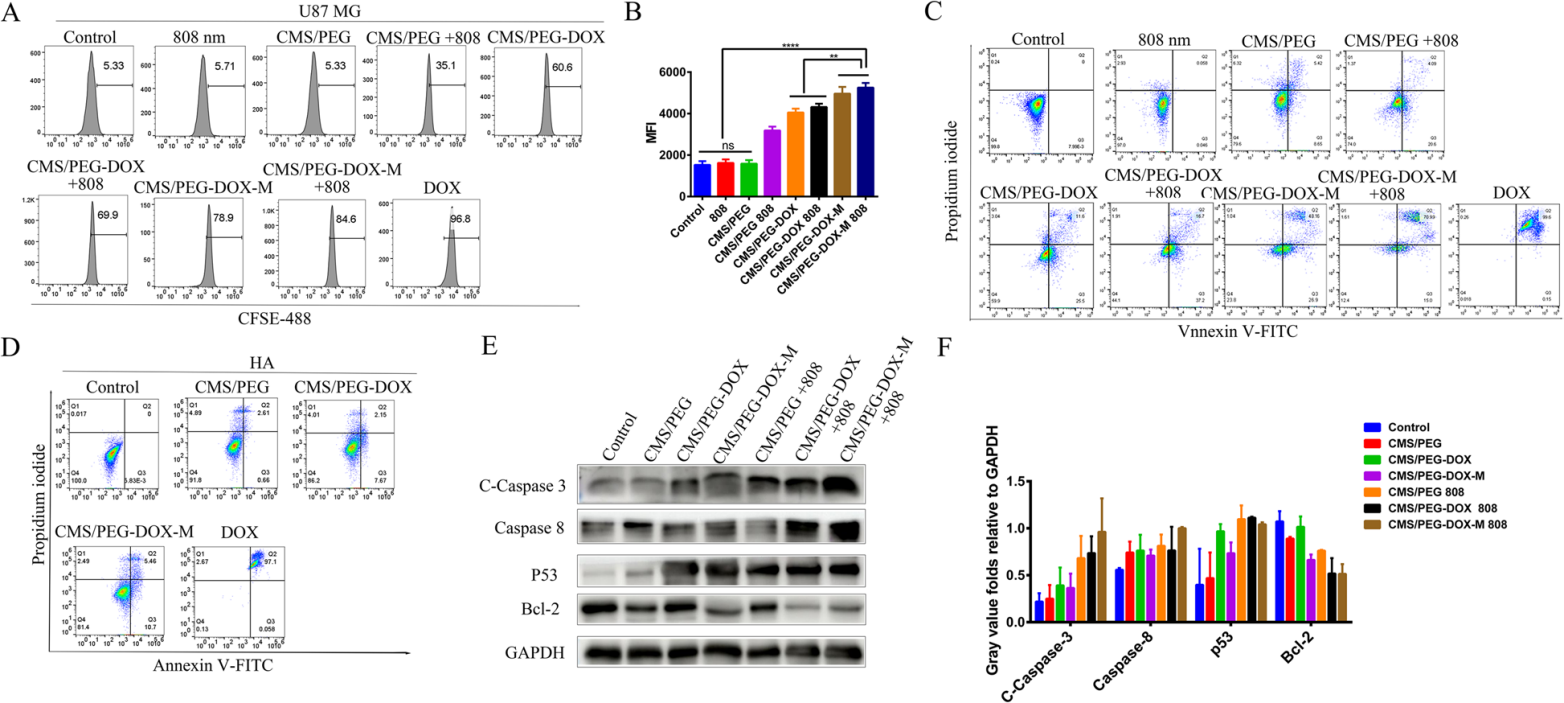
Cell proliferation assay, cell apoptosis assay, and Western blots of U87 MG and/or HA cells. **A** The cell proliferation assay analyzed by flow cytometry of U87 MG cells were treated with vehicle (Control group) or with either of the indicated nanodrugs with or without infrared laser irradiation (808 nm laser,1 W·cm^− 2^) for 24 h and labeled with CFSE. **B** MFI of CFSE in U87 MG detected by flow cytometry. **C**, **D**) Cells were treated with vehicle (Control group) or with either of the indicated nanodrugs with or without infrared laser irradiation (808 nm laser,1 W·cm^− 2^) for 24 h, and then stained with Annexin V-FITC and PI before being analyzed by flow cytometry for apoptosis/necrosis. Apoptosis of U87 MG cells (**B**) or apoptosis of HA cells (**C**). (Q1) necrotic cells, (Q2) late apoptotic cells, (Q3) early apoptotic cells, (Q4) viable cells. **E** Western blots of the expression levels of pro-apoptotic and anti-apoptotic proteins in U87 cells 24 h after either of the indicated treatment. (**F**) The grayscale values of the indicated protein bands on Western blots that were quantified and normalized to the expression level of the housekeeping gene, GAPDH, after the total cell lysates of U87 MG cells treated for 24 h from either of the treatment groups were resolved by SDS-PAGE. The data are based on three independent experiments. *p* values were calculated by Tukey’s post-test (**p < 0.01, ****p < 0.0001).CMS, Cu_2_MoS_4_; PEG, polyethylene glycol; DOX, doxorubicin; M, cell membrane of U87 MG cells; CFSE, carboxyfluorescein succinimide ester dye; FITC, fluorescein isothiocyanate; PI, propidium iodide

To examine whether the nanodrugs that we developed affect cell proliferation and cell apoptosis, U87 MG cells were labeled with CFSE. Treatment with free DOX, CMS/PEG-DOX-M, or CMS/PEG-DOX-M + 808 inhibited U87 MG cell proliferation as detected by flow cytometry (Fig. 5A-B). CMS/PEG-DOX-M had a more significant antiproliferative effect than CMS/PEG-DOX (Fig. 5A-B), due to a higher accumulation of DOX in U87 MG cells through actively targeted uptake (Fig. S8B, Supporting Information). Moreover, either CMS/PEG-DOX-M or CMS/PEG-DOX, after 808 nm laser irradiation, further inhibited the proliferation of U87 MG cells (Fig. 5A-B). In addition, in the apoptosis study, it was found that the CMS/PEG-DOX-M + 808 resulted in more than 85% of U87 MG cells undergoing apoptosis after 24 h of treatment, and the pro-apoptotic effect was higher than that of CMS/PEG-DOX-M (Fig. 5C), and CMS/PEG-DOX-M had a more significant effect than CMS/PEG-DOX (Fig. 5C). The nanodrugs further promoted apoptosis of U87 MG cells after irradiation with an 808 nm laser (Fig. 5C). The pro-apoptotic effect of CMS/PEG-DOX or CMS/PEG-DOX-M was consistent with the results of cell viability of the above (Fig. 3C-D). Interestingly, the CMS/PEG-DOX or CMS/PEG-DOX-M induced almost no apoptosis in HA cells (Fig. 5D), indicating that almost no DOX was released from CMS/PEG-DOX or CMS/PEG-DOX-M into HA cells, whereas free DOX caused apoptosis of almost all HA cells (Fig. 5D). These results show that the side effects of nanodrugs are much less severe, which is consistent with the previous experiments of cellular uptake (Fig. 4D). To examine the molecular mechanism of apoptosis induced by CMS/PEG-DOX or CMS/PEG-DOX-M, the expression levels of pro-apoptotic and anti-apoptotic proteins were detected by Western blotting. After drug treatment, the pro-apoptotic factor, Caspase 3, was activated, and the protein expression levels of cleaved Caspase 3 and Caspase 8 were increased (Fig. 5E-F). In contrast, the protein expression level of Bcl-2, an anti-apoptotic factor, was decreased, while the expression level of tumor suppressor gene P53 was increased (Fig. 5E-F). These results indicate that U87 MG cells underwent apoptosis after each of the different treatments and CMS/PEG-DOX-M induced more apoptotic cells, especially after 808 nm laser irradiation.

### Intracellular ROS measurement

To examine whether the DOX-loaded nanodrugs that we developed can induce the production of ROS in GBM cells like free DOX, it was found that CMS/PEG-DOX and CMS/PEG-DOX-M induced a 1.5-fold and 1.7-fold increase in ROS production in U87 MG cells within 24 h of nanodrug treatment, respectively, after laser irradiation (Fig. S9A-B, Supporting Information). Therefore, like free DOX, DOX released from CMS/PEG-DOX or CMS/PEG-DOX-M nanodrugs can also induce ROS production in GBM cells (Fig. S9A-B, Supporting Information). In conclusion, ROS-mediated DNA damage is considered to be one of the potential mechanisms by which DOX induces apoptosis in GBM cells.

### Cellular CRT induction assay

To examine whether nanodrugs that we developed can induce the externalization of CRT on cell membrane from cytoplasm, U87 MG cells were treated with each of the indicated nanodrugs, and then the level of membranous CRT was examined by flow cytometry. Both treatments promoted the level of membranous CRT in U87 MG cells (Fig. S9C-D, Supporting Information). Compared with CMS/PEG-DOX, a higher level of membranous CRT was observed in CMS/PEG-DOX-M cells, and the level of membranous CRT was increased from 42.9 to 67.3% in U87 MG cells induced with CMS/PEG-DOX-M (Fig. S9C, Supporting Information). The level of membranous CRT was further increased to 88.1% after laser irradiation (Fig. S9C-D, Supporting Information).

### Drug penetration of an in vitro BBB model

**Fig. 6:**
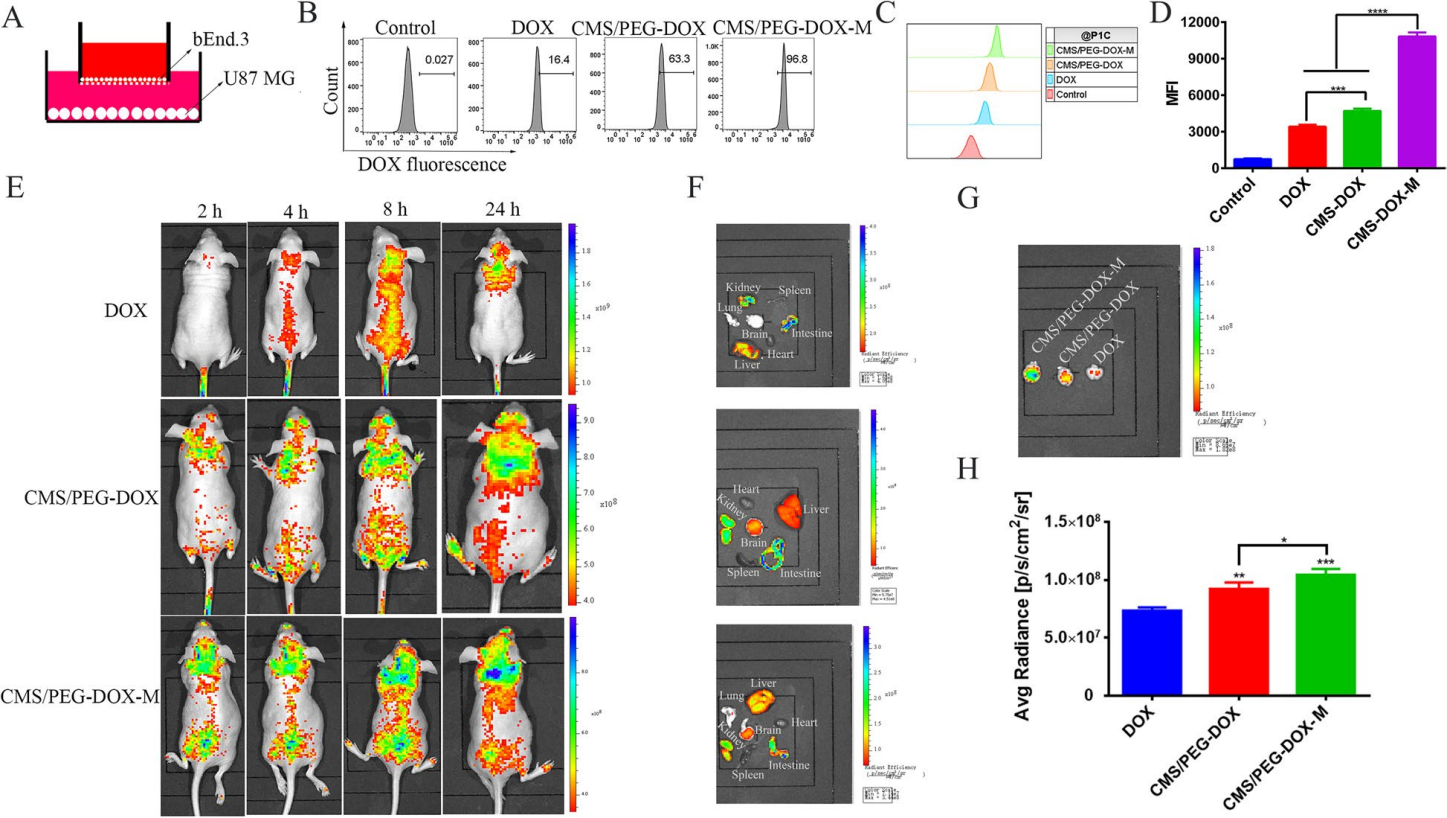
Detection of the BBB penetration of nanodrugs in the in vitro and in vivo models. **A** Schematic diagram of the in vitro BBB model of the co-cultured bEnd.3 and U87 MG cells. **B** The percentage of DOX uptake in U87 MG cells measured by flow cytometry after the co-cultured bEnd.3 cells were treated with either of the indicated treatments. **C**-**D** The profile of DOX uptake (**C**) and MFI of DOX (**D**) detected by flow cytometry in U87 MG cells after the co-cultured bEnd.3 cells were treated with either of the indicated treatments. **E** In vivo DOX fluorescence images obtained at the indicated time points of U87 MG-LUC tumor-bearing nude mice after tail vein administration of CMS/PEG-DOX-M, CMS/PEG-DOX, or DOX (CMS: 15 mg/kg, DOX: 10 mg/kg, 100 μL). **F** Average radiance of DOX fluorescence detected in mouse brain orthotopic glioma tissue. **G** Ex vivo DOX fluorescence images of the major organs (heart, lungs, liver, spleen, kidney, intestine, brain) surgically removed from the same mouse of each group at 24 h time point as seen in the panel C. (H) Ex vivo DOX fluorescence images of the mouse brains of the same mouse from each group at 24 h time point as seen in the panel D. BBB, blood-brain barrier; CMS, Cu_2_MoS_4_; PEG, polyethylene glycol; DOX, doxorubicin; M, cell membrane of U87 MG cells; MFI, mean fluorescence intensity; LUC, luciferase

To examine whether our nanodrugs can penetrate the BBB, we used bEnd.3, a mouse brain microvascular endothelial cell line, and U87 MG cells as an in vitro BBB model to test the permeability of nanoparticles that we developed (Fig. 6A). The drug uptake of U87 MG cells was measured by flow cytometry after 24 h of nanodrug treatment. Free DOX hardly entered into U87 MG cells through bEnd.3 cells (Fig. 6B-D). U87 MG cells took up only 16.4% of free DOX (Fig. 6B-D). In contrast, more than 60% of CMS/PEG-DOX entered U87 MG cells and released the loaded DOX drug (Fig. 6B-D). Almost all of CMS/PEG-DOX-M entered U87 MG cells, and more than 96% of the loaded DOX drug was released (Fig. 6B-D). Active cell targeting, efficient cellular uptake, and rapid drug release of CMS/PEG-DOX-M in U87 MG cells were again confirmed.

### Antitumor effects of nanodrugs in mouse tumor models

The reason for the poor efficacy of chemotherapy is that the drug fails to effectively penetrate the BBB to reach the tumor. First, we used fluorescence imaging to detect whether our nanodrugs can go through the BBB. The orthotopic xenograft mice of U87 MG cells were injected with CMS/PEG-DOX-M, CMS/PEG-DOX or DOX via tail vein. DOX fluorescence in mice was detected at different time points (2 h, 4 h, 8 h, or 24 h) (Fig. 6E), and DOX fluorescence in some isolated major organs was detected after 24 h (Fig. 6F). It was found that after injection of DOX, almost no fluorescence was seen in the mouse brain even 24 h later (Fig. 6E), and ex vivo tissue fluorescence imaging also showed that there was no DOX in the brain (Fig. 6F). DOX fluorescence was detected in the mouse brain within 2 h after the injection of each of two nanodrugs, and the fluorescence intensity gradually increased until 24 h (Fig. 6E), and the fluorescence intensity of injected CMS/PEG-DOX-M was significantly higher than that of CMS/PEG-DOX (Fig. 6E), and the highest amount of DOX was detected in the brain from the mice treated with CMS/PEG-DOX-M, but not with other treatments (Fig. 6G-H). These results demonstrate that free DOX can hardly penetrate the BBB, while CMS/PEG-DOX and CMS/PEG-DOX-M can effectively reach the tumor through the BBB to release the loaded DOX, and CMS/PEG-DOX-M has an active cell targeting ability so that more DOX is accumulated within the tumor.

**Fig. 7:**
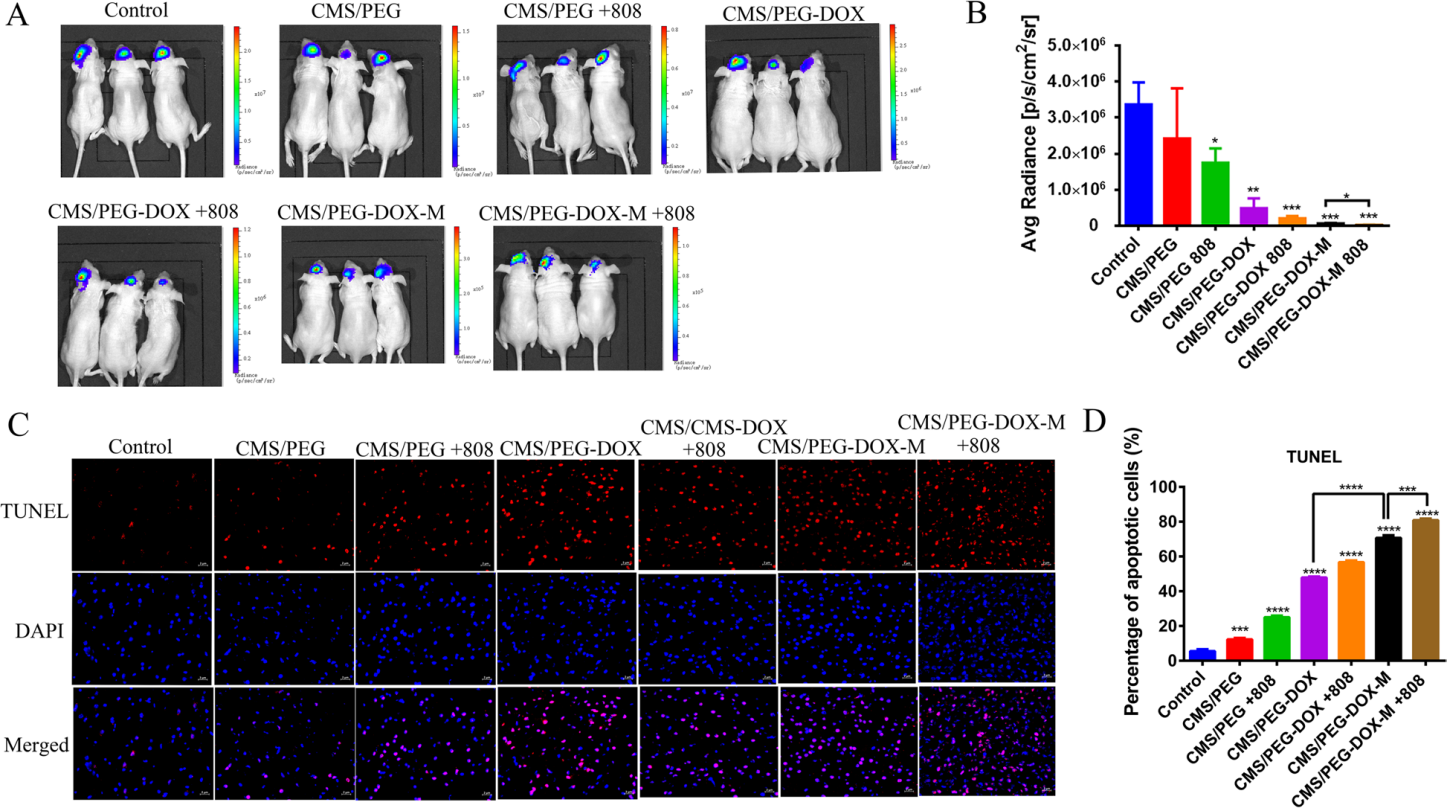
In vivo bioluminescent imaging and immunofluorescence staining of anti-tumor effect of nanodrugs in mouse orthotopic glioma model. **A** Firefly luciferase bioluminescent images of orthotopic U87 MG-Luc bearing nude mice after 15 days in the different groups. **B** Average radiance of luciferase bioluminescence detected in mouse brain orthotopic glioma tissue. **C** TUNEL stained images of the cryosections of mouse brain orthotopic glioma tissue from the different groups after 15 days of treatment. The scale bar is 2 μm. **D** Quantification of TUNEL assay results. LUC, Luciferase; TUNEL, Terminal-deoxynucleotidyl Transferase Mediated Nick End Labeling

To verify its photothermal effect, orthotopic glioma tumor-bearing mice were injected with CMS/PEG-DOX-M, CMS/PEG-DOX, or PBS via tail vein. After 24 h, mice were irradiated with the 808 nm laser, and the body temperature changes were monitored by an infrared thermal imager at different time points (1 min, 2 min, 3 min, 4 min, or 5 min) (Fig. S10A-B, Supporting Information). Compared with the other two treatments, the temperature of the tumor treated with CMS/PEG-DOX-M was raised to 45.0 °C within 5 min, causing most of the tumor tissue to be damaged (Fig. S10A-B, Supporting Information). In vivo treatment experiments, the orthotopic xenograft nude mice of U87 MG cells were divided into seven groups: vehicle group, CMS/PEG, CMS/PEG plus 808 nm laser irradiation (CMS/PEG + 808), CMS/PEG-DOX, CMS/PEG-DOX plus 808 nm laser irradiation (CMS/PEG-DOX + 808), CMS/PEG-DOX-M, and CMS/PEG-DOX-M plus 808 nm laser irradiation (CMS/PEG-DOX-M + 808). Among them, mice in groups 3, 5 and 7 were irradiated with 808 nm laser light for 5 min. In the next 15 days, the luciferase bioluminescence intensity of the mouse brain tumor in each group was imaged at the indicated days (Fig. S11A, Supporting Information) and on day 15, the luciferase bioluminescence intensity of three mouse brain tumors in each group was imaged (Fig. 7A) and quantified (Fig. 7B). Luciferase imaging clearly showed that CMS/PEG-DOX-M + 808 had the best antitumor efficacy (Fig. 7A), as evidenced by the quantification data (Fig. 7B). The mean bioluminescence intensity of three mouse brain tumors in CMS/PEG-DOX-M, CMS/PEG-DOX-M + 808, CMS/PEG-DOX, CMS/PEG-DOX + 808, CMS/PEG, CMS/PEG + 808, and control groups was 6 × 10^4^, 3 × 10^4^, 5 × 10^5^, 2 × 10^5^, 2.4 × 10^6^, 1.7 × 10^6^, and 3.4 × 10^6^ p/s/cm^2^/sr, respectively, after 15 days of treatment (Fig. 7B). CMS/PEG-DOX-M and CMS/PEG-DOX-M + 808 achieved a mean bioluminescence intensity that was one and two orders of magnitude lower, respectively, compared with CMS/PEG-DOX and CMS/PEG-DOX + 808 groups and the other groups, which are consistent with their small antitumor effects seen in the bioluminescence imaging data (Fig. 7A and Fig. S11A, Supporting Information). The mouse’s body weight was weighed every two days for 15 days (Fig. S11B, Supporting Information). No significant difference in mouse body weight was observed among all groups during the 15-day treatment period (Fig. S11B, Supporting Information), indicating that there was almost no systemic toxicity of the nanodrugs that we developed. Compared with the control group, TUNEL staining of tumor tissue sections showed that the other treatment groups had different degrees of pro-apoptotic effect. However, tissue sections of brain tumors treated with CMS/PEG-DOX-M + 808 showed maximum apoptosis (Fig. 7C-D). At the same time, immunofluorescence staining of CRT showed that compared with the control group, the other treatments caused the CRT originally inside the cells to translocate to the cell membrane (Fig. S11C, Supporting Information). Among all groups, the brain tumor tissue sections from CMS/PEG-DOX-M + 808 treatment group showed the highest level of membranous CRT (Fig. S11C, Supporting Information). In order to verify the apoptosis of tumor cells in tumor tissue, H &E staining was performed on the tumor tissue sections of mice with different treatment groups, and the results showed that the tumor cells in the tumor tissues of the nanodrug treated mice had solidified, fragmented, and blue-black nuclei, as well as the reddish cytoplasm, indicating that tumor cells underwent apoptosis (Fig. S12, Supporting Information). In addition, the histological analysis of the major organs was performed to further explore the biocompatibility of the nanodrugs (Fig. S12, Supporting Information). H & E stained images of the heart, liver, spleen, lung, and kidney from mice that underwent with a variety of treatments show negligible inflammation or damage, suggesting that these treatments did not cause noticeable toxicity in vivo. These results show that CMS/PEG-DOX-M has good biocompatibility and potential for clinical translation.

## Discussion

In this study, we developed a biomimetic nanodrug CMS/PEG-DOX-M that can effectively go through the BBB to achieve multimodal treatment of glioma. First of all, we chose CMS as the backbone of nanomaterials and then made CMS into a mesoporous nanosphere as a carrier for chemotherapeutic drugs for the reasons below. Firstly, CMS is a powerful and safe nanomaterial that can be designed in multiple shapes. For example, CMS was able to eradicate drug-resistant bacteria as nanoplates [28]. CMS effectively inhibited mouse subcutaneous tumors as a hollow nanosphere carrier with a size greater than 100 nm [23]. Secondly, once the CMS is absorbed into the tumor, it can produce ·OH by Fenton-like reaction and deplete GSH overexpressed in TME, thus reducing the anti-oxidation capacity of the tumor to rebalance the TME. Thirdly, the catalase-like property of CMS can allow CMS to react with endogenous H_2_O_2_ to form O_2_ under the condition of anoxic TME, alleviating the anoxic condition of TME. Fourthly, CMS has a good photothermal property of the near-infrared absorption effect and is an excellent producer of cytotoxic superoxide anion (·O_2_^−^) under 808 nm laser irradiation [23]. However, CMS has not been studied in intracranial orthotopic glioma models. In order to make the CMS easily go through the BBB, we optimized the synthesis method by changing the synthetic conditions and constructed a mesoporous nanosphere material with a smaller size of much less than 100 nm and larger cavities. Our synthesis method did not affect the performance of the CMS/PEG nanospheres. In fact, CMS itself showed a remarkable ability of phototherapeutic tumor inhibition observed by modulating the TME in this study.

Subsequently, in order to improve its dispersion, biocompatibility, and half-life, CMS was modified by PEG-4000. To further potentiate the function of CMS/PEG itself and achieve multimodal functionalities, DOX was loaded into CMS/PEG nanoparticles to make CMS/PEG-DOX nanodrug. DOX was steadily and gradually released from CMS/PEG-DOX nanoparticles under acidic conditions, which are the characteristics of the TME, suggesting that CMS/PEG-DOX nanodrugs that we developed will most likely work the same way in an in vivo glioma model. In contrast, almost no DOX was released into normal cells, which almost did not cause apoptosis of normal cells, suggesting that this nanodrug, if used in vivo, will generate much less severe side effects than that of free DOX. DOX-induced ROS production in GBM cell lines has been reported [29]. Both DOX released from CMS/PEG-DOX or CMS/PEG-DOX-M nanodrug, and CMS can produce a large amount of exogenous ROS. An excessive level of endogenous ROS in tumor cells makes them more sensitive to additional exogenous ROS, so the effect of exogenous ROS is more likely to lead to tumor cell death than normal cells [30].

The number of nanoparticles that finally reach the target tissue is generally low because most of the nanoparticles get trapped in the reticuloendothelial system and cleared by the immune system due to the size and immunogenicity of nanoparticles. Certain proteins expressed on the surface of tumor cell membranes, such as PD-L1 and CXCR4, are known to help nanoparticles evade the immune system [31]. To further enhance the combined antitumor effects of CMS and DOX, while minimizing the side effects of free DOX, the biomimetic nanodrug, CMS/PEG-DOX-M, was developed by encapsulating CMS/PEG-DOX nanodrug with U87 MG cell membranes and tested in an orthotopic glioma model. Our results show that this biomimetic nanodrug has a highly efficient BBB penetration capacity, an improved tumor targeting ability, and an enhanced release of chemotherapy drug, while modulating the TME, thereby achieving a higher accumulation of DOX and promoting a higher number of apoptotic glioma cells in the orthotopic glioma tissue. In other words, this biomimetic nanodrug synergistically inhibits the growth of orthotopic glioma in a multimodal manner by combining chemotherapy and photothermal therapy.

Studies have shown that the TME-regulating ability of nanodrugs is beneficial to antitumor immunity and promotion of macrophage polarization [32]. Enhanced chemotherapy and photothermal therapy also allow apoptosis or necrotic tumor cells to release tumor-associated antigens [33], which would be recognized and engulfed by antigen-presenting cells such as dendritic cells (DCs), to induce antitumor cellular immunity. CRT expression level is an important indicator of immune activation and therapeutic effect. The membranous exposure of CRT in tumor cells releases an “eat me” signal and stimulates phagocytosis of these tumor cells by DCs and phagocytes [31, 34]. Therefore, externalized CRT can promote the antigen presentation and maturation of DCs, and induce further activation of immune cells to enhance tumor-killing ability. Nanodrugs were reported to promote CRT’s externalization from the cytoplasm of tumor cells, which further enhances antigen presentation and maturation of immune cells, thereby regulating local immune processes [31]. In this work, we found that CMS/PEG-DOX-M is able to translocate a high level of CRT from cytoplasm to cell membrane in glioma cells, suggesting that cell membrane wrapping not only enhances the targeting ability of nanodrugs to glioma tissue by evading the immune system, but also promotes the activation of local immunity in the tumor tissue.

Our future work will focus on the combination therapy of nanodrugs and immunity, such as cytokine changes, immune cell polarization, and cytotoxic T lymphocyte (CTL) infiltration. Overall, this treatment system holds great potentials for the clinical application of nanodrugs in treating GBM, other central nervous systems (CNS) tumors, and even tumors of different types.

## Conclusion

**Fig. 8:**
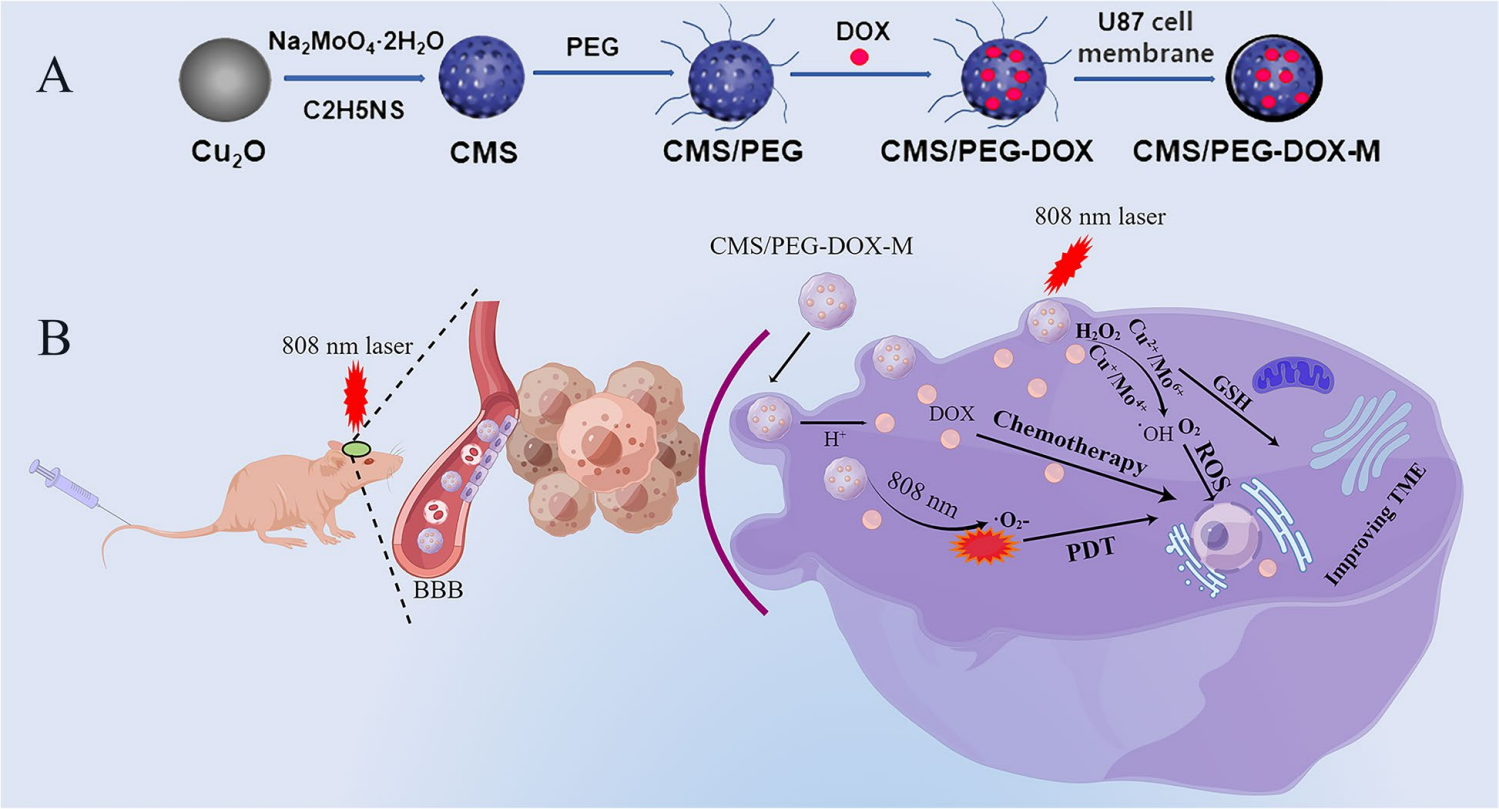
(**A**) Schematic diagram of the synthesis route of CMS/PEG-DOX-M of nanodrug. **B** Schematic diagram of nanodrug in the near-infrared laser-mediated multi-modal synergistic treatment of glioblastoma. CMS, Cu_2_MoS_4_; PEG, polyethylene glycol; DOX, doxorubicin; M, cell membrane of U87 MG cells; BBB, blood-brain barrier; PDT, photodynamic therapy; ROS, reactive oxygen species; GSH, glutathione; TME, tumor microenvironment

We successfully constructed a multifunctional biomimetic nanoparticle delivery system with a smaller nanoparticle size, high drug load, and good homology-targeting ability. It has been demonstrated, for the first time, that this biomimetic nanodrug can effectively cross the BBB and achieve a great multimodal therapeutic efficacy of gliomas in vivo and in vitro by combining photothermal therapy and chemotherapy (Fig. 8). This biomimetic nanodrug system holds great potentials in the treatment of brain tumors and may be extended to the delivery of a variety of anti-tumor drugs for the treatment of extracranial tumors.

## Supplementary Information

**Additional file 1.** The online version contains supplementary material available online.

## Data Availability

All data generated or analyzed during this study are included in this published article.
