# Supplementary material for: Efficiently targeted therapy of glioblastoma xenograft via multifunctional biomimetic nanodrugs
Source: Biomater Res. 2022 Dec 2;26:71. doi: 10.1186/s40824-022-00309-y (PMC9717509; doi:10.1186/s40824-022-00309-y)
Supplement: Supplementary file 1 — Additional file 1. The online version contains supplementary material available online. [file 40824_2022_309_MOESM1_ESM.docx]

**Supporting Information**

**Efficiently targeted therapy of glioblastoma xenograft via multifunctional biomimetic nanodrugs**

**Supporting Experimental Section**

**Materials and Reagents**

Copper (II) chloride dihydrate (CuCl_2_·2H_2_O), sodium hydroxide (NaOH), absolute ethanol, and hydrogen peroxide were purchased from Sinopharm (Beijing, China). Hydrochloric acid (HCl) was obtained from Nanjing Chemical Reagent Company. Polyvinylpyrrolidone (PVP) and methylene blue (MB) were purchased from Wo kai (Shanghai, China). Ascorbic acid (AA), sodium molybdate dihydrate (Na_2_MoO_4_·2H_2_O, 99%), glutathione (GSH, 98%), 2-nitrobenzoic acid (DTNB, ≥98%) and glucose were purchased from Aladdin (Shanghai, China). Ethylene glycol was purchased from Greagent (Shanghai, China). PEG-4000 and 1,3-diphenylisobenzofuran (DPBF) were obtained from Sigma (Darmstadt, Germany). Thioacetamide (C_2_H_5_NS, ≥99%) was purchased from Energy Chemical (Shanghai, China). All the above purchased chemical reagents were used directly without further purification.

**Synthesis of Hollow Cu_2_MoS_4_ Nanoparticles**

Hollow mesoporous CMS nanospheres were prepared in two steps. First, Cu_2_O nanospheres were prepared, and then Cu_2_O nanospheres were used as a sacrificial template^[1]^. In brief, 0.171 g CuCl_2_·2H_2_O and 3.333 g PVP were dispersed in 100 mL deionized water and magnetically stirred for 10 min until completely dissolved by magnetic stirring. Then, 10 mL of 2 M NaOH and 10 mL of 0.6 M ascorbic acid were quickly added, while magnetic stirring was maintained. After continuing the magnetic stirring for 1 h, the product was collected by centrifugation at 13000 rpm for 5 min, and washed three times with deionized water and ethanol, respectively. Finally, the product was dried overnight at 60 °C in a vacuum oven. The product Cu_2_O nanoparticles were obtained. The prepared 40 mg Cu_2_O nanoparticles and 120 mg glucose were dissolved in 20 mL ethylene glycol by ultrasound. The added glucose prevented the structural collapse of the hollow mesoporous nanospheres of CMS^[2]^. Then, 70 mg Na_2_MoO_4_·2H_2_O and 120 mg C_2_H_5_NS were added to the sonicated solution under ultrasound treatment for 30 min. the mixture was transferred to a 45 mL polytetrafluoroethylene lined stainless steel autoclave and held for 24 hours at 200 °C. The product was centrifuged at 10,000 rpm for 5 min and washed three times with deionized water and ethanol, respectively, followed by drying overnight at 60°C in a vacuum oven. The product Cu_2_MoS_4_（CMS）nanoparticles were obtained. PEG-4000 has the function of improving the dispersion and compatibility of the material and overcoming the immunogenicity of the host immune system^[3]^.Then, 5 mg of the prepared CMS was dissolved in 50 mL of deionized water, and 5 mg of PEG-4000 was added to the CMS solution to form CMS/PEG under magnetic stirring, then the CMS/PEG was collected by centrifugation, washed with deionized water three times, and stored until use.

**Characterizations**

Transmission electron microscopy (TEM, Talos F200X, acceleration voltage = 200 KV, point resolution = 0.25 nm) was applied to characterize the morphology and size of nanoparticles. The X-ray diffraction (XRD) patterns were tested with a D8-Discover diffractometer (Bruker, Germany). The X-ray photoelectron spectra (XPS) were taken on a Thermo ESCALAB 250 XI electron spectrometer using Mono Kα (1486.6 eV) as the excitation source (Thermo, US). According to XPS, the elemental composition of the CMS was analyzed^[4]^. UV–vis spectra were measured with a Shimadzu UV 2600 UV–vis spectrophotometer (Shimadzu, Japan). Zeta potential and dynamic light scattering (DLS) were determined by a Malvern Zetasizer system (Nano ZS, Malvern, UK). Surface area and pore size were obtained by measuring nitrogen adsorption/desorption curve analysis using Micromeritics TriStar II 3 flex equipment (Micromeritics, US). Thermal images were recorded using a Seek Thermal Camera and Seek Thermal application (Seek Thermal, US). Inductively Coupled Plasma Mass Spectrometry (ICP-MS) was taken on a NexION1000G of PerkinElmer (Shanghai, China).

**Determination of oxygen production performance of CMS**

CMS has catalase-like activity and can react with H_2_O_2_ to produce O_2_. At room temperature, 100 µg·mL^-1^ CMS (1 mL) was added to 100 µM H_2_O_2_ (1 mL) solution of different pH values (5.5, 6.5, or 7.4) with magnetic stirring for different durations of time. The changes of UV characteristic absorption peak of H_2_O_2_ were characterized by UV-Vis spectra in different duration of time (0, 10, 20, 30, 40, 50, or 60 min). The UV absorbance of H_2_O_2_ and the variation of the bubbles formed in the solution were used to reflect the formation of O_2_ and to verify the catalase like activity of CMS.

**Detection of extracellularly produced hydroxyl radical ·OH by CMS**

CMS can generate ·OH through Fenton-like reactions^[5]^. ·OH is the main oxidant for the removal of methylene blue (MB)^[6]^. Therefore, MB was used as an indicator to detect the ·OH generated during the oxidation reaction. 20 mg CMS was added to 3 mL of 4 μg·mL^-1^ MB solution with different pH values (5.5, 6.5, or 7.4) under magnetic stirring for 30 minutes in the dark. Then 100 µL of 3 mM hydrogen peroxide was added to the mixed solution. After stirring for different durations of time (0, 5, 10, or 20 min), CMS was removed by centrifugation from the mixed solution. The change in the UV absorbance of MB at 660 nm and the color of the solution reflect the degree of ·OH generation.

**Detection of extracellular GSH consumption by CMS**

CMS has glutathione peroxidase-like activity. 240 µL of DTNB-PBS solution (3 µg·mL^-1^) of different pH (5.5, 6.5, or 7.5) and 30 µL of 10 mM GSH aqueous solution were added to 100 µL CMS aqueous solution of different concentrations (0, 25, 50, 100, or 200 µg·mL^-1^). The mixture was magnetically stirred for 1 h at room temperature, then the CMS was removed by centrifugation, and the absorbance of the supernatant was determined by a UV-Vis spectrophotometer. The change in the UV absorbance of GSH and the color of the solution reflect the degree of GSH consumption.

**Detection of extracellularly generated ·O_2_^-^ by CMS**

CMS can generate cytotoxic superoxide anion (·O_2_^-^) under 808 nm laser irradiation. 1,3-Diphenylisobenzofuran (DPBF) was used as a probe to detect the production of extracellular ·O_2_^-^. 10 µL of DPBF solution (10 mg·mL^-1^) was added to 1 mL CMS aqueous solution (50 µg·mL^-1^). Then it was irradiated for a different duration of time (0, 3, 6, 9, 12, 15, or 20 min) under 808nm laser (1W·cm^-2^). Then the CMS was removed by centrifugation, and the UV-Vis absorption spectra of DPBF in the supernatant at 420 nm were detected. The change in absorbance of DPBF reflects the degree of ·O_2_^-^ formation.

**Examinations of photothermal effect and stability of CMS aqueous solution**

1 mL CMS aqueous solution with a different concentration (0, 50, 100, 150, or 200 µg·mL^-1^) was irradiated by 808 nm laser (1W·cm^-2^). The temperature was measured by an infrared camera in a real-time manner, and the temperature change was recorded every 30 seconds for 5 min. In addition, its photothermal stability was measured by cyclic laser irradiation. In brief, the CMS aqueous solution (200 µg·mL^-1^) was irradiated by an 808 nm laser (1W·cm^-2^) for 5 min. Then the laser was turned off. The aqueous solution was naturally cooled for 5 min, and the temperature of the aqueous solution was recorded by an infrared camera every 30 seconds. The above process was repeated for 4 times.

**Cytotoxicity assay**

The cytotoxicity of nanodrugs was assessed by MTT assay. In brief, 200 μL of U87 MG cells were seeded in a 96-well plate at a density of 5 × 10^4^ cells/well and incubated overnight in the incubator. The medium was replaced with fresh medium containing nanoparticles at a different concentration (0, 3.125, 6.25, 12.5, 25, 50, 100, or 200 µg/mL) and cells were incubated for 24 hours. Cells were washed once with PBS after the medium was aspirated, then 200 μL of MTT solution (0.5 mg/mL) was added to each well before cells were incubated for 4 hours in the incubator. Finally, 150 μL of DMSO was added to each well after the MTT solution was aspirated, and followed by the absorbance measurement at 490 nm with a universal microplate reader. Cell viability was determined by (A_test_-blank/A_control_-blank) × 100%.

**Cellular Uptake of CMS/PEG-DOX and CMS/PEG-DOX-M Assay**

Cellular uptake of CMS/PEG-DOX-M was used to evaluate the tumor cell targeting ability and drug release ability in specific sites of biomimetic nanodrugs. Briefly, HA and U87 MG cells were inoculated at a density of 1×10^5^ cells into a confocal laser scanning microscope (CLSM) plate and 6-well plate, respectively and cultured overnight. CMS/PEG-DOX-M (25 μg/ml, 2 μL) was added to each well at different time points (2h, 4h, 6h, or 8h), the cells were then fixed in 4% paraformaldehyde solution for 10 min, followed by nuclei staining with DAPI dye solution for 10 min, and due to imaging of DOX red auto-fluorescence. Finally, cell uptake and release of biomimetic nanodrugs were observed using a confocal laser scanning microscope (CLSM) plate, and cell uptake and release were quantified using flow cytometry (Navios, Beckman).

***In vitro* cell apoptosis and proliferation detection**

U87 MG cells were inoculated into a 6-well culture plate at a density of 5×10^5^ cells per well and cultured in 5% CO_2_ at 37°C for 12 h. Afterwards, U87 cells were treated with vehicle, CMS/PEG, 808 nm laser irradiation (808 nm), CMS/PEG +808, CMS/PEG-DOX, CMS/PEG-DOX +808, DOX, CMS/PEG-DOX-M, or CMS/PEG-DOX-M +808. 4 hours after drug addition, cells were irradiated with 808 nm laser (1W·cm^-2^) for 5 min, and then cultured for an additional 24 h. After the cells were collected by trypsin digestion, the cells were washed twice with cold PBS and treated according to the Annexin V-FITC and PI apoptosis detection kit (KGA108, Keygen). Finally, the apoptosis of U87 MG cells was examined by flow cytometry (Navios, Beckman). Furthermore, to study the effects of various treatments on cell proliferation, U87 MG cells were incubated with fluorescent carboxyfluorescein succinimide ester dye (CFSE, 2.5 µM) for 20 min in an incubator, each cell division, the amount of CFSE in cells decreased, resulting in a sequential halving of cellular fluorescence intensity with each mitotic event^[7]^, then after the medium was removed, the cells were washed and treated with free DOX, CMS/PEG-DOX, or CMS/PEG-DOX-M for 24 h, followed by the medium replacement for 24 h. After the cells were collected by trypsin digestion, the cells were washed twice with cold PBS. Finally, the proliferation of U87 MG cells was examined by using flow cytometry (Navios, Beckman).

**Detection of externalized calreticulin (CRT) on cell membrane from the cytoplasm by flow cytometry**

U87 MG cells were seeded into a 6-well culture plate at a density of 5×10^5^ cells per well and cultured in 5% CO_2_ at 37 °C for 12 h. Afterwards, U87 MG cells were treated with vehicle, CMS/PEG, 808 nm, CMS/PEG +808, CMS/PEG-DOX, CMS/PEG-DOX +808, DOX, CMS/PEG-DOX-M, or CMS/PEG-DOX-M +808. 4 hours after drug addition, cells were irradiated with 808 nm laser (1W·cm^-2^) for 5 minutes and then further cultured for 24 h. After the cells were collected by trypsin digestion and washed twice with ice-cold PBS, cells were loaded with Alexa Fluor 488 anti-CRT antibody (ab196158, Abcam) and incubated at 4℃ for 30 min, then washed three times with ice-cold PBS. Finally, the CRT fluorescence was measured by flow cytometry (Navios, Beckman).

**Detection of intracellular reactive oxygen species (ROS) production**

By 2',7'-dichlorofluorescein diacetate (DCFH-DA) as a cell membrane permeable dye to detect the production of ROS in vitro^[8]^. U87 MG cells were seeded into a 6-well culture plate at a density of 5×10^5^ cells per well and cultured in 5% CO_2_ at 37 °C for 12 h. Afterwards, U87 MG cells were treated with vehicle, 808 nm, CMS/PEG,

CMS/PEG +808, CMS/PEG-DOX, CMS/PEG-DOX +808, DOX, CMS/PEG-DOX-M, or CMS/PEG-DOX-M +808. 4 hours after drug addition, cells were irradiated with 808 nm laser (1W·cm^-2^) for 5 minutes, and then cultured for 24 h. After the cells were collected and washed twice with ice-cold PBS, the DCFH-DA (MCE) was added to cells that were treated with each different treatment above and the mixture was incubated at room temperature for 20 min. Finally, the cells were washed three times with PBS. The fluorescence was detected by flow cytometry (Navios, Beckman).

***In vitro* BBB-GBM co-culture model**

The blood-brain barrier (BBB) consists primarily of endothelial cells lining brain capillaries and is characterized by the presence of tight junctions and an efflux transport system^[9]^. bEnd3 cells are an immortalized mouse cerebral microvascular endothelial cell line, similar to primary mouse endothelial cells, its permeability and expression of the tight junction protein claudin-5 are similar to that of primary mouse endothelial cells, which is a suitable BBB cell culture model. Studies have shown that this cellular model is ideal for testing the permeability of nanoparticles^[10]^, and so they can be used to mimic BBB^[11]^. U87 MG cells were seeded at a density of 5×10^4^/cm^2^ in the lower chamber of the transwell insert (pore size 0.4 μm, diameter 6.5 mm, 24 wells, Corning, USA), and after the cells were adhered, bEnd3 cells were seeded at a density of 8×10^4^/cm^2^ in the upper chamber of the transwell insert to generate a co-culture model. DOX, CMS/PEG-DOX (25 μg/ml, 2 μl), or CMS/PEG-DOX-M were added to bEnd3 cells in the upper chamber, and the drug intake in U87 MG cells was measured by flow cytometry (Navios, Beckman) after 24h drug treatment.

**Western blot analysis**

The expressions of Caspase-8, cleaved Caspase-3, P53, and Bcl-2 proteins were determined by Western blotting. Total protein was extracted from differently treated U87 MG cells using RIPA lysis buffer and separated by 10% SDS-PAGE, then transferred to polyvinylidene difluoride (PVDF) membrane. The membrane was blocked with 5% skim milk at room temperature for 1 hour, and then incubated with Caspase-8 (1:3000, ab32125, Abcam), cleaved Caspase-3 (1:500, ab32042, Abcam), Bcl-2(1:2000, ab182858, Abcam), P53 (1:1000, ab32389, Abcam), or GAPDH (1:2500, ab9485, Abcam) overnight at 4°C. The membrane was incubated with secondary antibody HRP conjugated goat anti-rabbit IgG (1:5000, ab181662, Abcam) at room temperature for 1 h. Finally, chemiluminescence signals of the target proteins were developed using ECL detection kit (KeyGen Biotch) and visualized and analyzed using ImageQuant 800 (GE, USA).

**H&E Staining**

Frozen mouse brain tissue sections were fixed with 4% paraformaldehyde for 15 min and washed three times in PBS. The hematoxylin staining solution was stained for 8 min, rinsed in water for 5 min to remove excess staining solution, differentiated in differentiation solution for 20s, and treated with the anti-blue solution for 1 min. Eosin staining solution staining for 1 min, wash in water for about 30s, 70% ethanol for 10s, 80% ethanol for 10s, 90% ethanol for 10s, and anhydrous ethanol for 10s. Xylene transparent for 5 min. Switch to fresh xylene and transparent for another 5 minutes. Seal the tablets with neutral gum. Observed under an upright microscope (Carl Zeiss Axio Scope Al, Germany).

**References**

[1] a)M. Chang, Z. Hou, M. Wang, M. Wang, P. Dang, J. Liu, M. Shu, B. Ding, A. A. Al Kheraif, C. Li, J. Lin, *Small* **2020**, 16, e1907146; b)C. C. Yec, H. C. Zeng, *J. Mater. Chem. A* **2014**, 2, 4843.

[2] a)M. Chang, M. Wang, M. Wang, M. Shu, B. Ding, C. Li, M. Pang, S. Cui, Z. Hou, J. Lin, *Adv Mater* **2019**, 31, e1905271; b)W. Zhen, S. An, W. Wang, Y. Liu, X. Jia, C. Wang, M. Zhang, X. Jiang, *Nanoscale* **2019**, 11, 9906.

[3] S. Dong, J. Xu, T. Jia, M. Xu, C. Zhong, G. Yang, J. Li, D. Yang, F. He, S. Gai, P. Yang, J. Lin, *Chem Sci* **2019**, 10, 4259.

[4] a)W. Chen, H. Chen, H. Zhu, Q. Gao, J. Luo, Y. Wang, S. Zhang, K. Zhang, C. Wang, Y. Xiong, Y. Wu, X. Zheng, W. Chu, L. Song, Z. Wu, *Small* **2014**, 10, 4637; b)K. Zhang, Y. Zheng, Y. Lin, C. Wang, H. Liu, D. Liu, C. Wu, S. Chen, Y. Chen, L. Song, *Phys Chem Chem Phys* **2016**, 19, 557; c)P. D. Tran, M. Nguyen, S. S. Pramana, A. Bhattacharjee, S. Y. Chiam, J. Fize, M. J. Field, V. Artero, L. H. Wong, J. Loo, J. Barber, *Energy & Environmental Science* **2012**, 5; d)S.-n. Li, R.-x. Ma, C.-y. Wang, *International Journal of Minerals, Metallurgy, and Materials* **2018**, 25, 310; e)J. Shan, K. Yang, W. Xiu, Q. Qiu, S. Dai, L. Yuwen, L. Weng, Z. Teng, L. Wang, *Small* **2020**, 16, e2001099; f)B. B. Chen, D. K. Ma, Q. P. Ke, W. Chen, S. M. Huang, *Phys Chem Chem Phys* **2016**, 18, 6713.

[5] X. Qian, J. Zhang, Z. Gu, Y. Chen, *Biomaterials* **2019**, 211, 1.

[6] G. Son, D. H. Kim, J. S. Lee, H. I. Kim, C. Lee, S. R. Kim, H. Lee, *J Environ Manage* **2018**, 206, 77.

[7] S. M. Kaech, R. Ahmed, *Nat Immunol* **2001**, 2, 415.

[8] Y. Liu, Z. Song, Y. Liu, X. Ma, W. Wang, Y. Ke, Y. Xu, D. Yu, H. Liu, *Acta Pharm Sin B* **2021**, 11, 1513.

[9] H. Liu, J. Zhang, X. Chen, X. S. Du, J. L. Zhang, G. Liu, W. G. Zhang, *Nanoscale* **2016**, 8, 7808.

[10] a)S. Tilloy, V. Monnaert, L. Fenart, H. Bricout, R. Cecchelli, E. Monflier, *Bioorg Med Chem Lett* **2006**, 16, 2154; b)M. Norouzi, V. Yathindranath, J. A. Thliveris, B. M. Kopec, T. J. Siahaan, D. W. Miller, *Sci Rep* **2020**, 10, 11292; c)S. Yang, S. Mei, H. Jin, B. Zhu, Y. Tian, J. Huo, X. Cui, A. Guo, Z. Zhao, *Plos One* **2017**, 12, e0187017.

[11] T. Watanabe, S. Dohgu, F. Takata, T. Nishioku, A. Nakashima, K. Futagami, A. Yamauchi, Y. Kataoka, *Biol Pharm Bull* **2013**, 36, 492.

**Supplementary Figures**

**
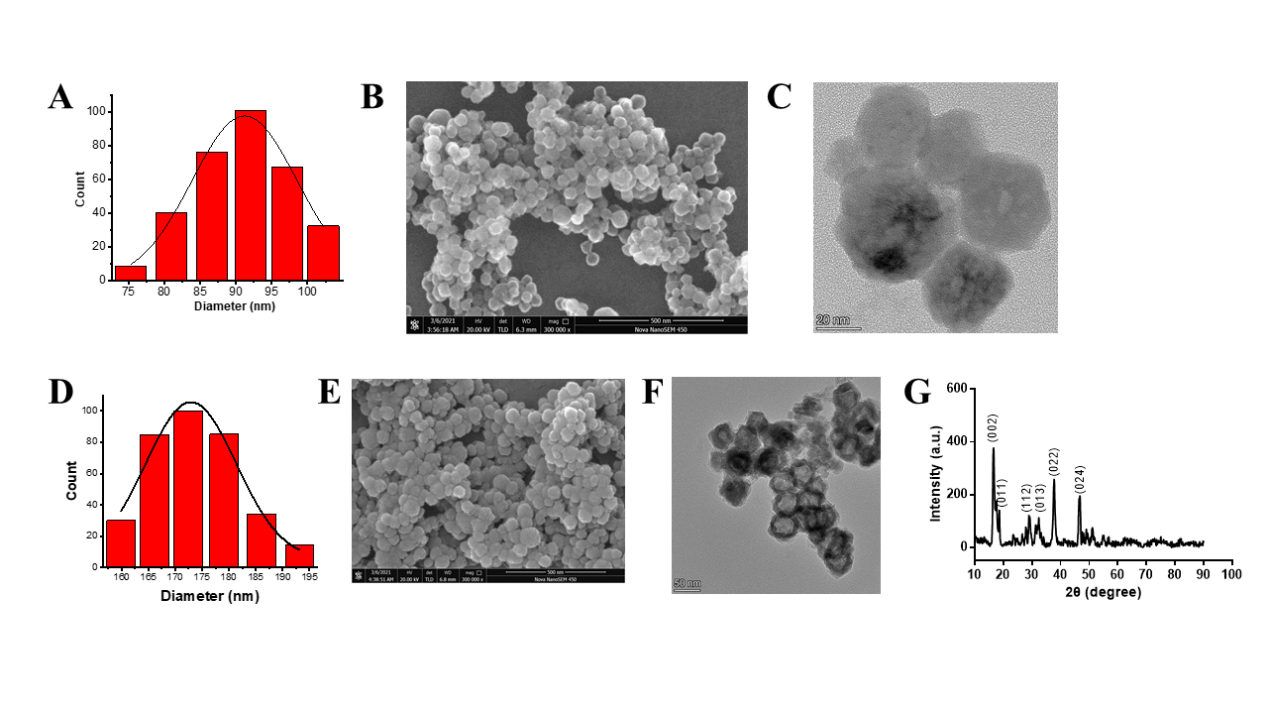
**

Figure S1. The measurements of the shape and size of Cu_2_O and CMS by DLS, SEM, TEM, and XRD. (A) The size distribution of Cu_2_O is measured by DLS in water. (B) The SEM image of Cu_2_O. (C) TEM image of Cu_2_O. (D) The size distribution of CMS was measured by DLS in water. (E) The SEM image of CMS. (F) TEM image of CMS. (G) The XRD pattern of CMS. CMS, Cu_2_MoS_4;_ DLS, dynamic light scattering; SEM, scanning electron microscopy; TEM, transmission electron microscopy; XRD, X-ray diffraction.


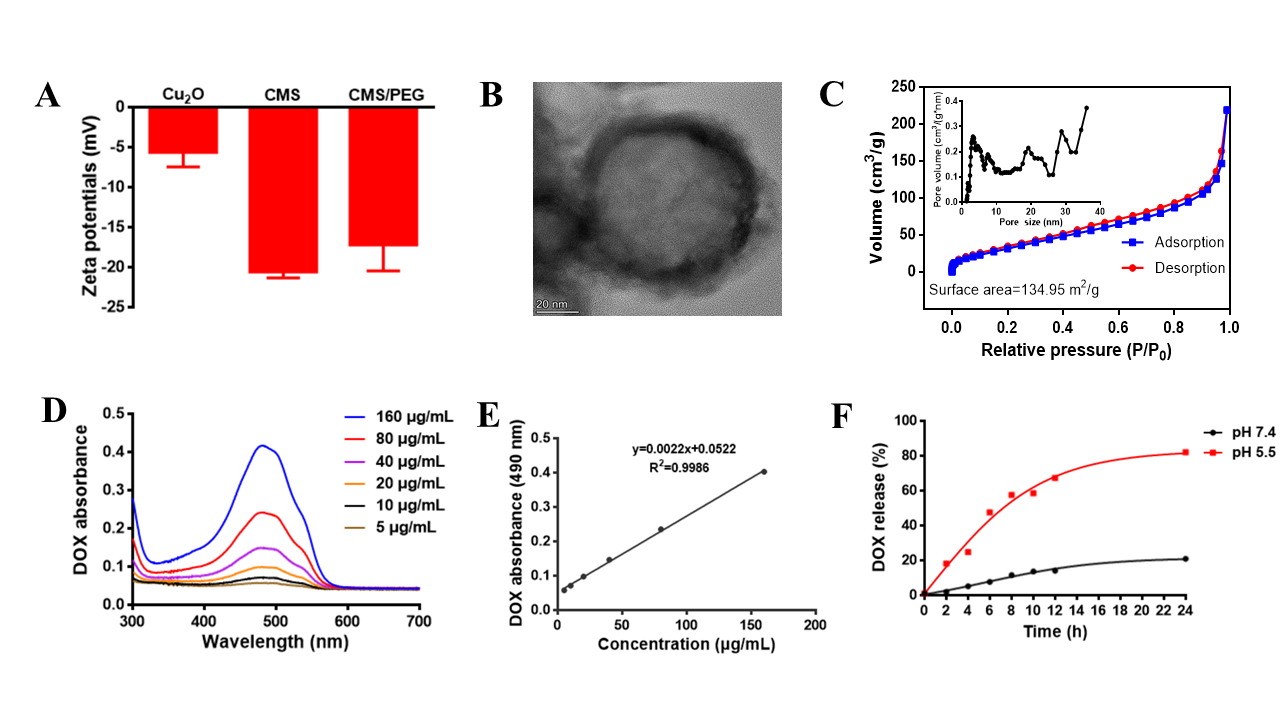


Figure S2. Measurements of Zeta potential, pore size, and DOX release of nanoparticles at different pH. (A) The Zeta potential of Cu_2_O, CMS, and CMS/PEG. (B) TEM image of a CMS/PEG nanoparticle. (C) The pore size distribution and N_2_ adsorption-desorption isothermal curves (inset) of CMS/PEG. (D) UV-Vis absorption curves of free DOX at different concentrations. (E) Standard curve of free DOX with six concentrations measured at the wavelength of 496 nm. (F) Percentage of DOX release from CMS/PEG-DOX solutions of the two different pH values at different time points. CMS, Cu_2_MoS_4_; PEG, polyethylene glycol; DOX, doxorubicin; TEM, transmission electron microscopy.


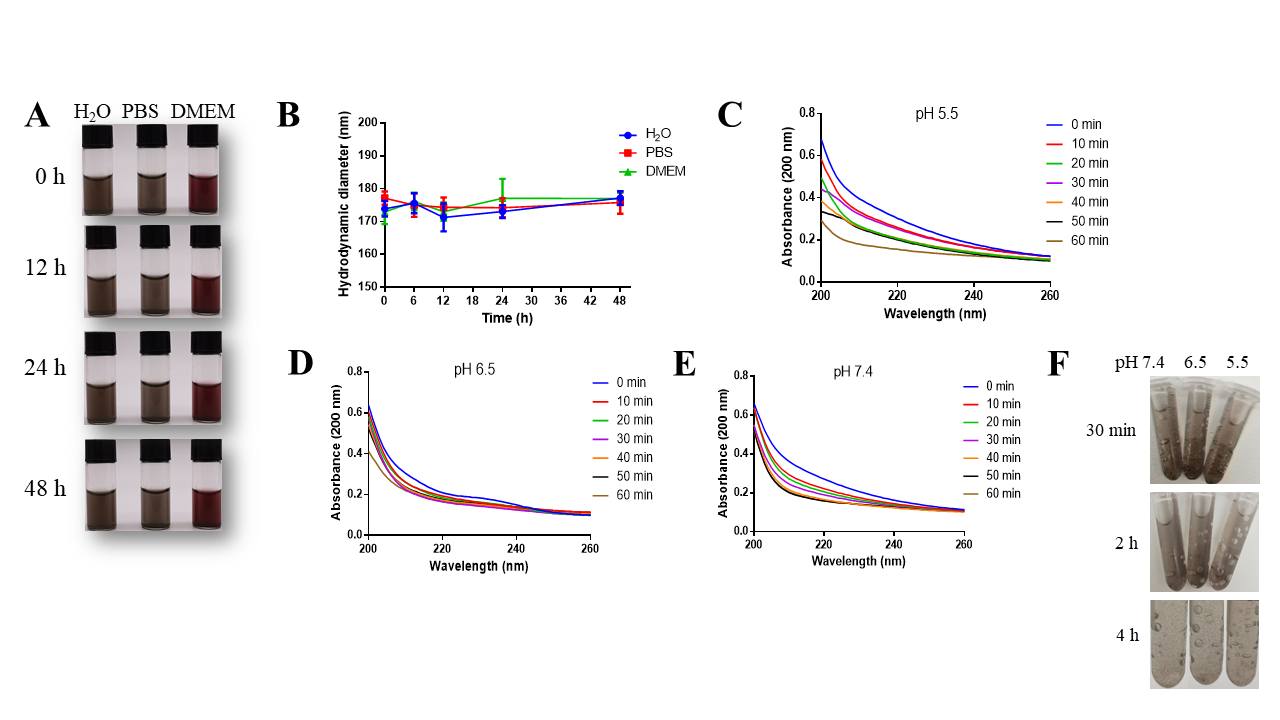


Figure S3. Stability of CMS/PEG nanoparticles suspended in different solutions and the measurement of the catalase-like activity of CMS/PEG nanoparticles. (A) The time-dependent state changes of CMS/PEG nanoparticles dispersed in H_2_O, PBS, or cell culture medium (DMEM). (B) The hydrodynamic diameters of CMS/PEG nanoparticles dispersed in H_2_O, PBS, or DMEM at different time points. The UV-Vis absorption curves of H_2_O_2_ in the supernatant after the exogenous H_2_O_2_ was added to the CMS/PEG nanoparticles dispersed in H_2_O at pH 5.5 (C), 6.5 (D), or 7.4 (E) over time. (F) The observed bubbles generated after the exogenous H_2_O_2_ was added to the CMS/PEG nanoparticles dispersed in H_2_O at pH 5.5, 6.5, or 7.4 at the indicated time points. CMS, Cu_2_MoS_4_; PEG, polyethylene glycol; DMEM, Dulbecco's Modified Eagle Medium.


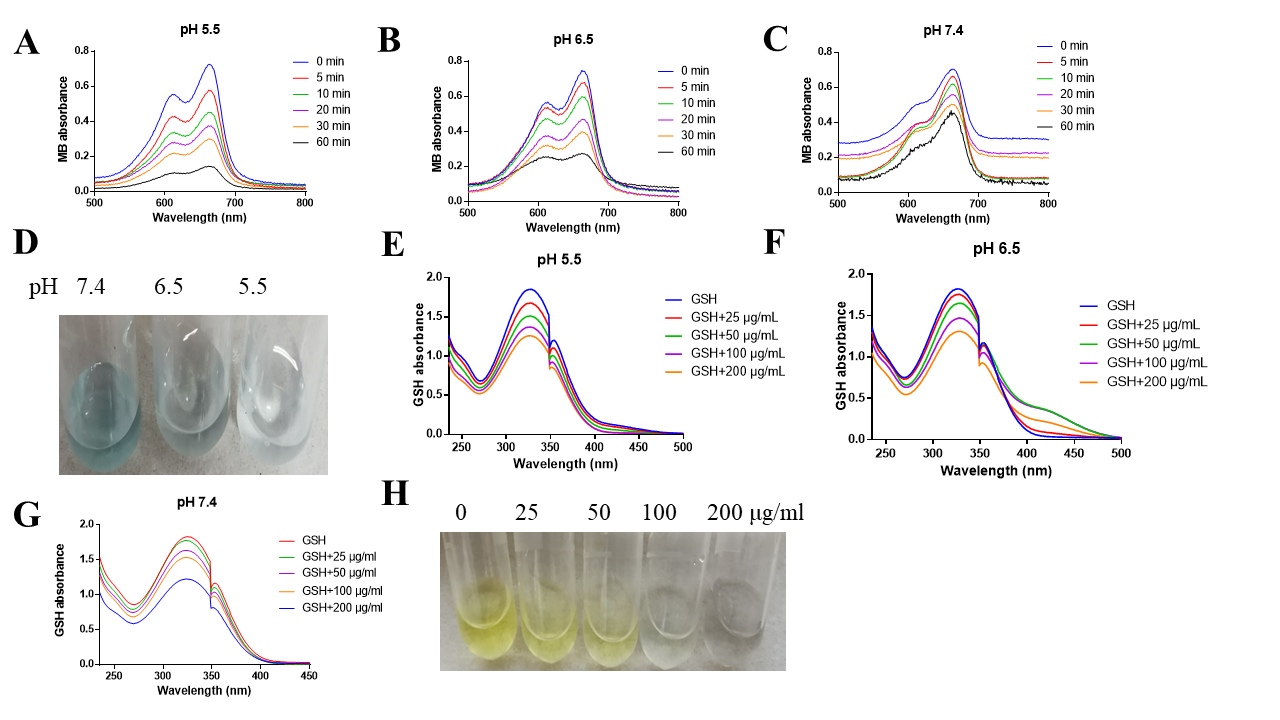


Figure S4. The detections of ·OH production and glutathione peroxidase-like activity of CMS/PEG by UV-Vis. (A-C) After adding MB and H_2_O_2_, the UV-Vis absorption curves of MB in the supernatants that reacts with ·OH are generated from the reaction of added H_2_O_2_ with the CMS/PEG solution of pH 5.5 (A), 6.5 (B), or 7.4 (C) over time. (D) The color changes of the supernatant of CMS/PEG solutions after H_2_O_2_ and MB were added to CMS/PEG solution of pH 5.5, 6.5, or 7.4. (E-G) The UV-Vis absorption curves of GSH in the supernatants after adding GSH to CMS/PEG solutions of different concentrations and pH 5.5 (E), 6.5 (F), and 7.5 (G). (H) The color changes of the consumed GSH were added to the supernatants of CMS/PEG with different concentrations. CMS, Cu_2_MoS_4_; PEG, polyethylene glycol; GSH, glutathione; MB, methylene blue.


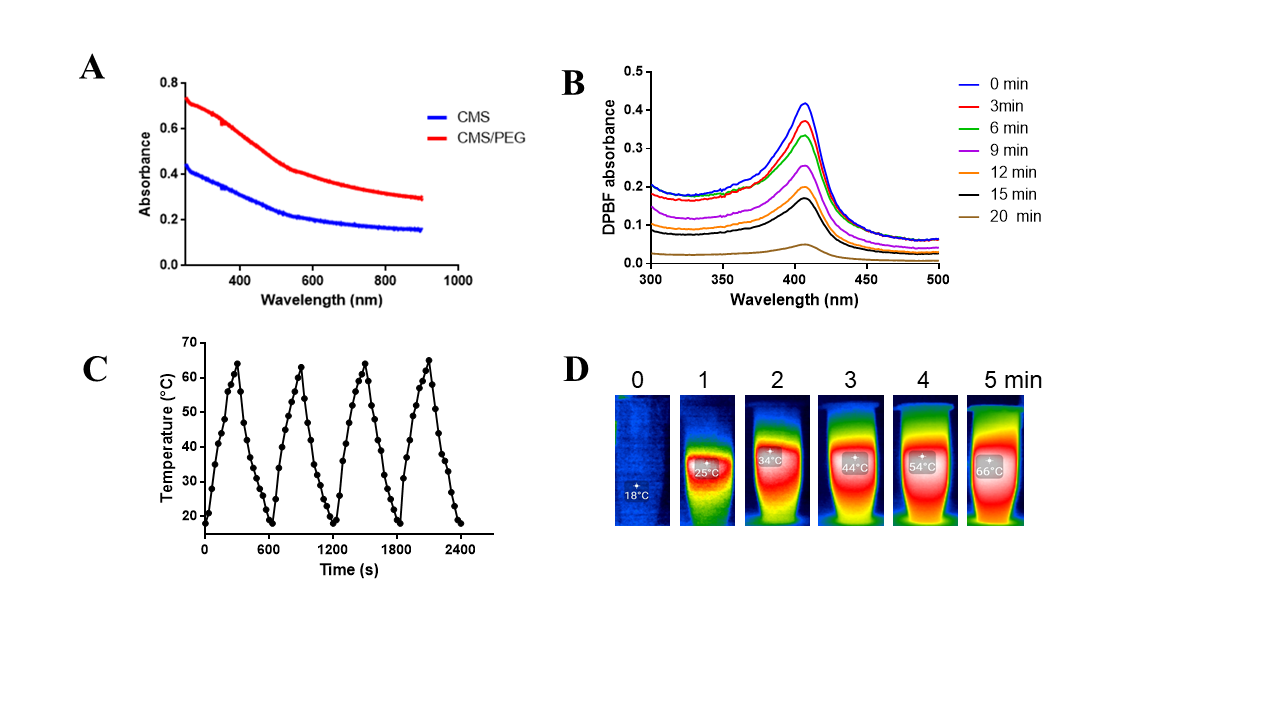


Figure S5. The adsorption spectra of CMS/PEG and DPBF, as well as photo-thermal conversion properties of CMS/PEG irradiated by 808 nm laser. (A) UV-Vis-NIR absorption spectra of CMS (1 mg/ml) and CMS/PEG (2 mg/ml). (B) UV-Vis absorption spectra of the unconsumed DPBF with ·O_2_^-^ in the supernatants of CMS/PEG solutions after DPBF was added to CMS/PEG dispersed in water (2 mg/ml) under infrared laser irradiation (808 nm, 1W·cm^−2^) for the indicated durations of time. (C) The photothermal stability of CMS/PEG was examined by cyclic heating (300 s) and cooling (300 s) steps under infrared laser irradiation (808 nm, 1W·cm^−2^). (D) Temperature changes of CMS/PEG under infrared laser irradiation (808 nm, 1W·cm^−2^) at different time points. NIR, near-infrared region; CMS, Cu_2_MoS_4_; PEG, polyethylene glycol; DPBF, 1,3-diphenylisobenzofuran.


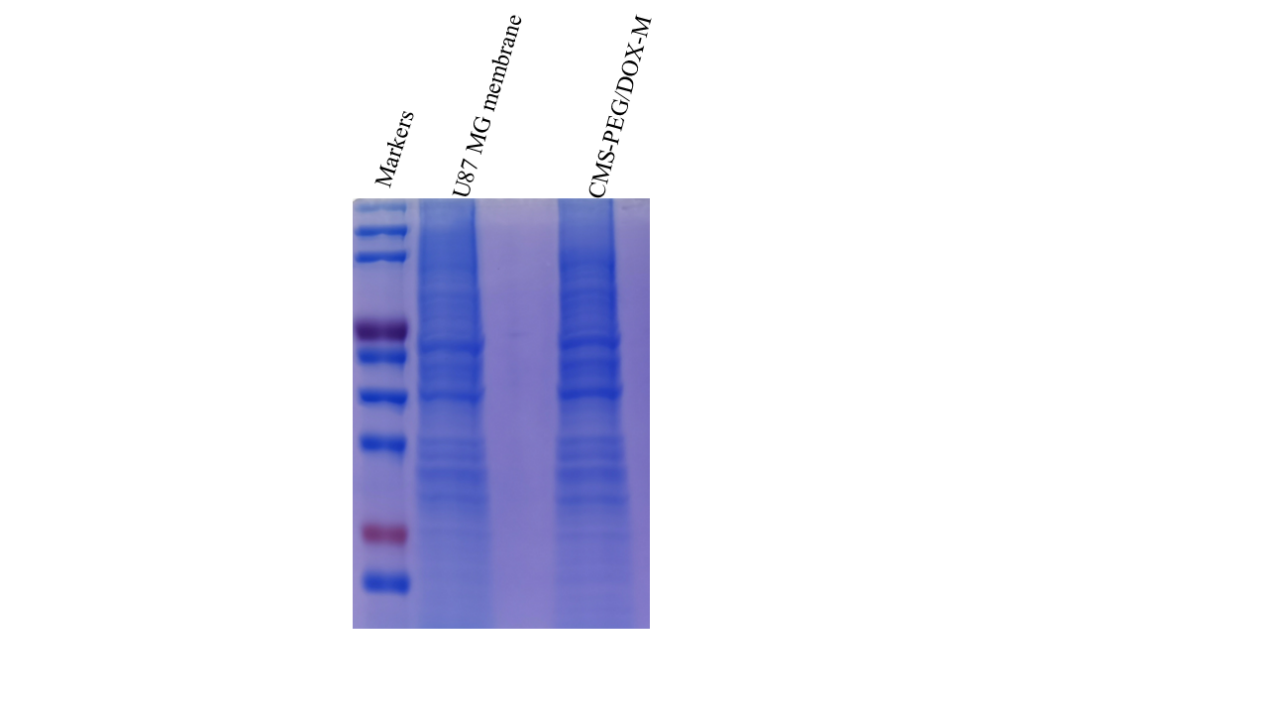


Figure S6. The SDS-PAGE profiles of the cell membrane proteins of U87 MG cells alone and CMS/PEG-DOX-M membrane proteins. Note: The third lane from the left is an empty lane. CMS, Cu_2_MoS_4_; PEG, polyethylene glycol; DOX, doxorubicin; M, the cell membrane of U87 MG cells; DOX, doxorubicin; SDS-PAGE, sodium dodecyl sulfate polyacrylamide gel electrophoresis.


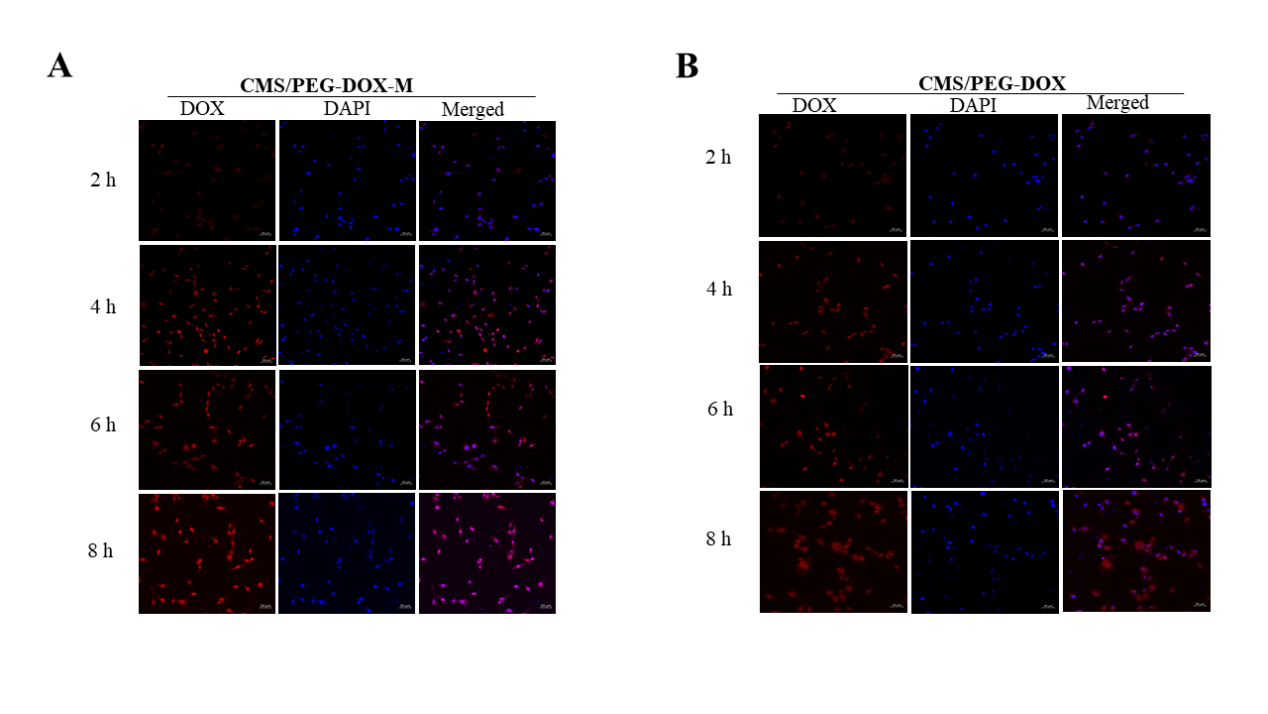


Figure S7. Examinations of the uptake of CMS/PEG-DOX-M or CMS/PEG-DOX into U87 MG and HA cells by confocal microscopy and flow cytometry. (A, B) Confocal images of U87 MG cells treated with CMS/PEG-DOX-M (A) or CMS/PEG-DOX (B) at the indicated time points (scale bar 50 μm). Blue and red colors represent DAPI and DOX fluorescence, respectively. The data are based on three independent experiments. p values were calculated by Tukey's post-test (**p<0.01, ***p<0.001, ****p<0.0001). CMS, Cu_2_MoS_4_; PEG, polyethylene glycol; DOX, doxorubicin; M, cell membrane of U87 MG cells; DOX, doxorubicin; DAPI, 4',6-diamidino-2-phenylindole; MFI, mean fluorescence intensity.


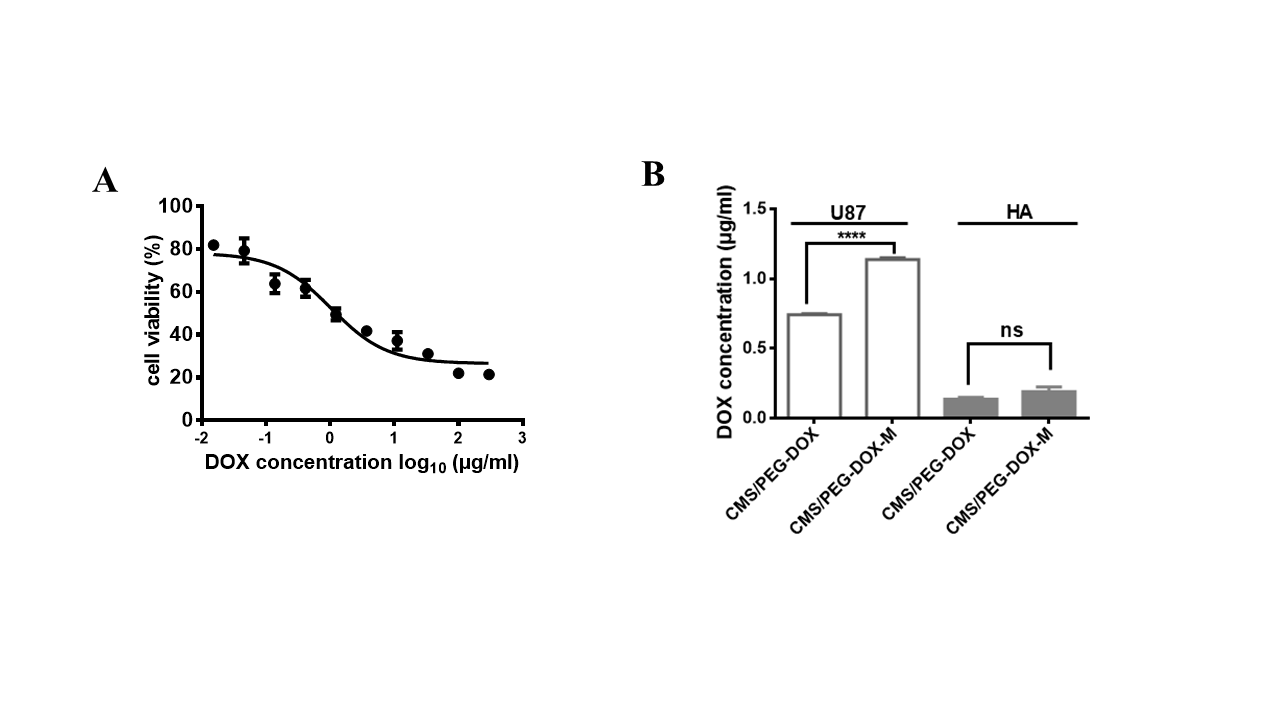


Figure S8. The IC50 of DOX and the amount of nanoparticle-mediated DOX uptake. (A) The IC50 of free DOX to U87 MG cells was measured by the MTT method. (B) The amount of DOX uptake was measured by its autofluorescence to U87 MG or HA cells treated with CMS/PEG-DOX or CMS/PEG-DOX-M nanoparticles for 24 h and then underwent cell lysis. The data are based on three independent experiments. p values were calculated by Tukey's post-test (**p<0.01, ****p<0.0001). CMS, Cu_2_MoS_4_; PEG, polyethylene glycol; DOX, doxorubicin; M, cell membrane of U87 MG cells; MFI, mean fluorescence intensity; CFSE, carboxyfluorescein succinimide ester dye; MTT, 3-(4,5-Dimethylthiazol-2-yl)-2,5-diphenyltetrazolium bromide; SDS-PAGE, sodium dodecyl sulfate-polyacrylamide gel electrophoresis.


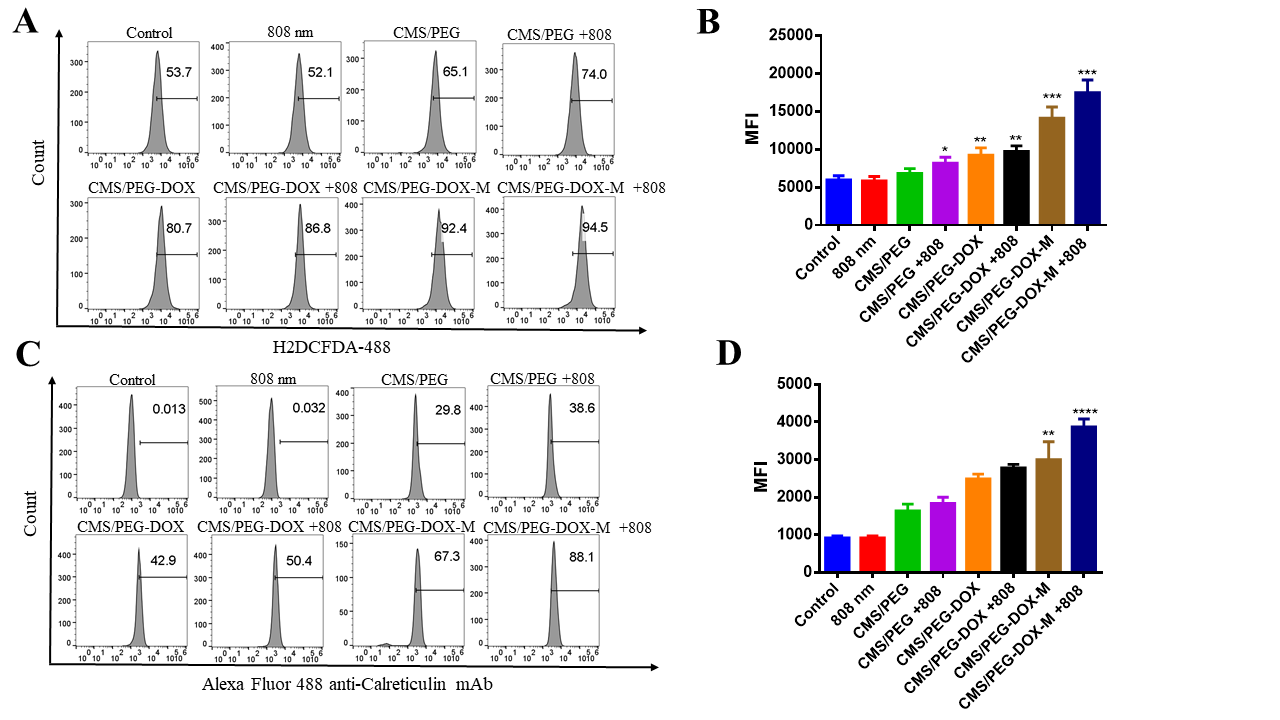


Figure S9. The production of ROS and membranous translocation of CRT induced by different treatments in U87 MG cells. (A-B) Flow cytometry images (A) showing the production of ROS and MFI of DCFH-DA (B) detected by flow cytometry in U87 MG cells with either of the indicated treatments. (C-D) Flow cytometry images (C) showing the membranous translocation of CRT and MFI of membranous CRT (D) detected by flow cytometry of U87 MG cells with either of the indicated treatments. The data are based on three independent experiments. p values were calculated by Tukey's post-test (*p<0.05, **p<0.01, ***p<0.001, ****p<0.0001). ROS, reactive oxygen species; CRT, Calreticulin; DCFH-DA, 2',7'-dichlorofluorescein diacetate; MFI, mean fluorescence intensity.


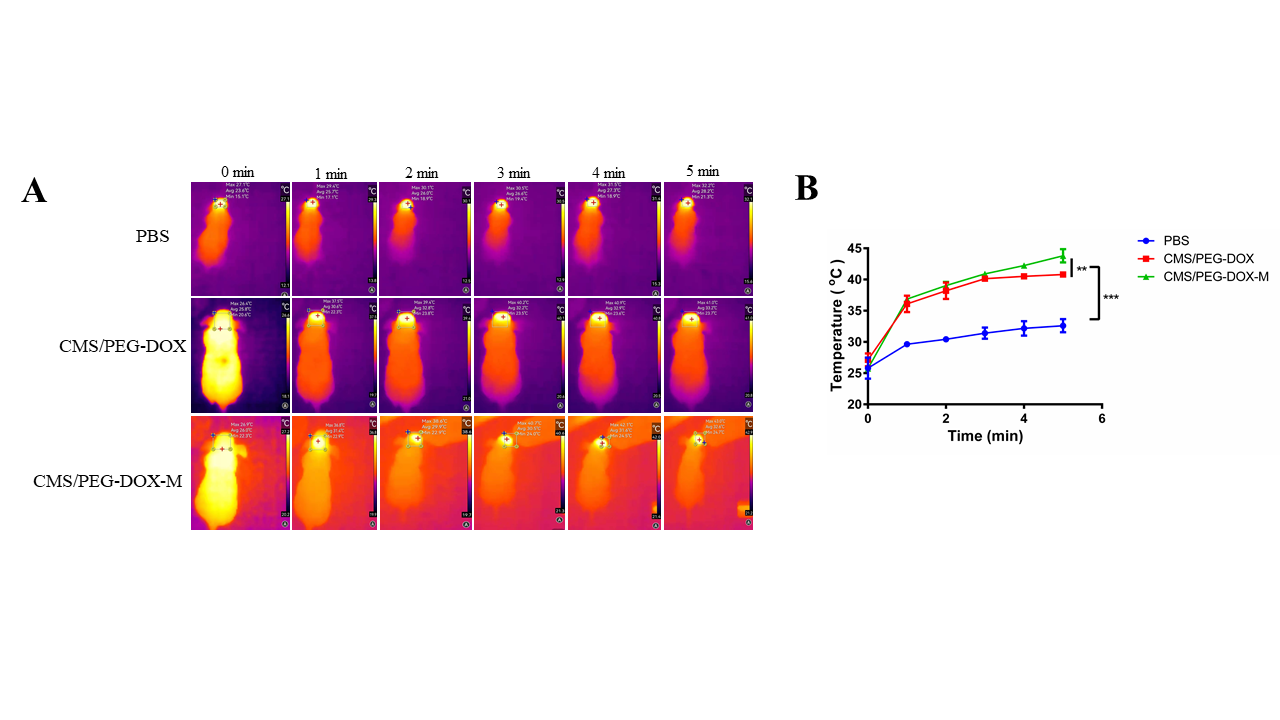


Figure S10. Detection of the BBB penetration of nanodrugs in the in vitro and in vivo models and examination of photothermal properties of nanodrugs. (A-B) The thermal images (A) and curves of body temperature changes (B) of U87 MG-LUC cancer-bearing mice taken at the indicated time points after tail vein injection with PBS, CMS/PEG-DOX-M, or CMS/PEG-DOX (100 μL, CMS: 15 mg/kg, DOX: 5 mg/kg) under the 808 nm irradiation laser (1w cm^-2^). CMS, Cu_2_MoS_4_; PEG, polyethylene glycol; DOX, doxorubicin; M, the cell membrane of U87 MG cells; BBB, blood-brain barrier; MFI, mean fluorescence intensity; LUC, luciferase.


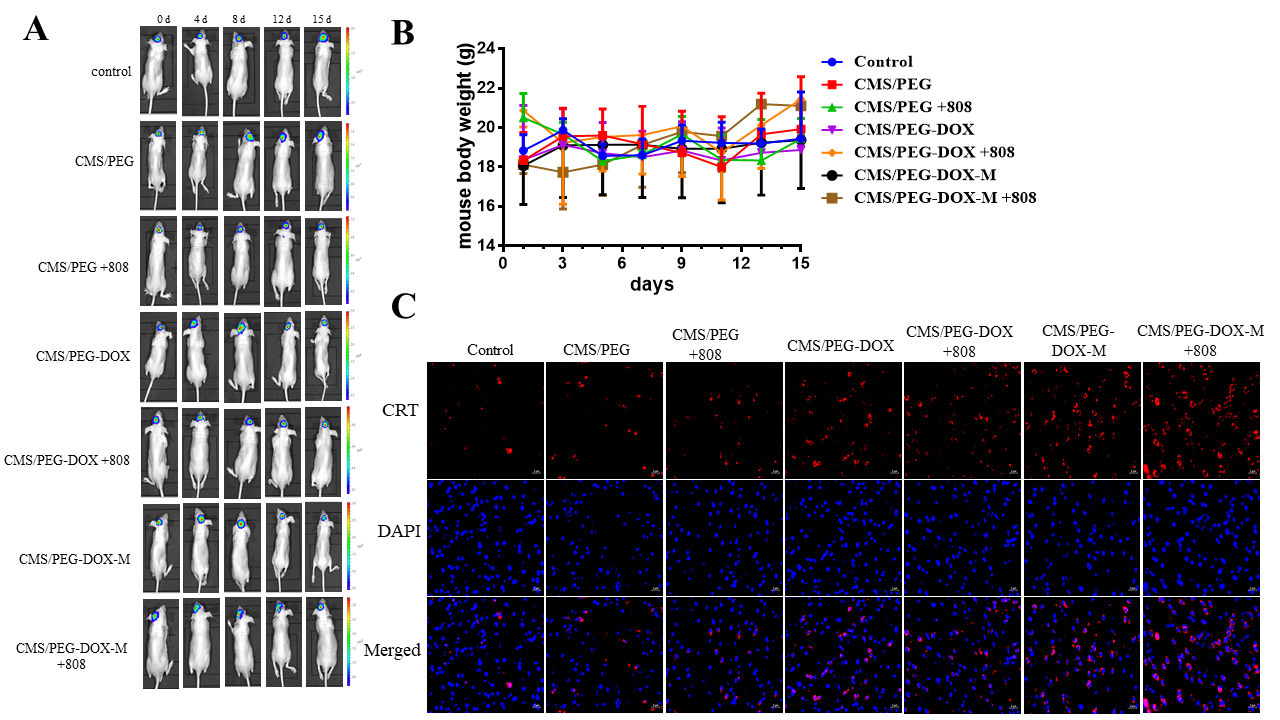


Figure S11. In vivo bioluminescent imaging, mouse body weight changes, and immunofluorescence staining of anticancer effect of nanodrugs in mouse orthotopic glioma model. (A) Firefly luciferase bioluminescent images of orthotopic U87 MG-LUC bearing nude mice in the indicated groups on the day (day 0) before the intravenous injection of nanodrugs and on days 4, 8, 12, and 15 after tail vein injection of nanodrugs. (B) Changes in body weight of mice in different treatment groups within 15 days of treatment. (C) Immunofluorescence staining images of CRT in the cryosections of mouse brain orthotopic glioma tissue from the different groups after 15 days of treatment (scale bar 2 μm). LUC, luciferase; CRT, Calreticulin; DAPI, 4',6-diamidino-2-phenylindole.


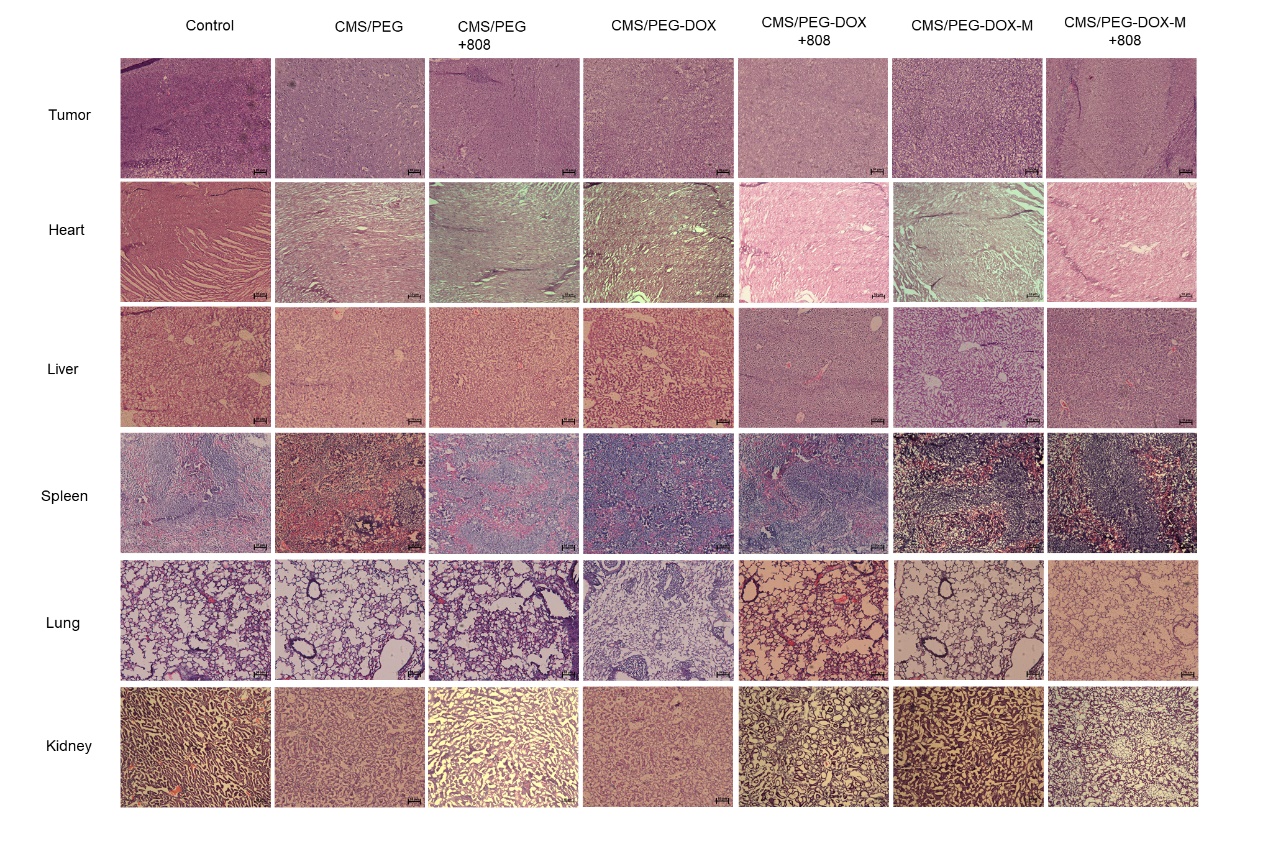


Figure S12. H &E stained images of tumors and major organs (heart, liver, spleen, lung, kidney) of mice in different treatment groups (scale bar 10 μm).
